# Canine *NAPEPLD*-associated models of human myelin disorders

**DOI:** 10.1038/s41598-018-23938-7

**Published:** 2018-04-11

**Authors:** K. M. Minor, A. Letko, D. Becker, M. Drögemüller, P. J. J. Mandigers, S. R. Bellekom, P. A. J. Leegwater, Q. E. M. Stassen, K. Putschbach, A. Fischer, T. Flegel, K. Matiasek, K. J. Ekenstedt, E. Furrow, E. E. Patterson, S. R. Platt, P. A. Kelly, J. P. Cassidy, G. D. Shelton, K. Lucot, D. L. Bannasch, H. Martineau, C. F. Muir, S. L. Priestnall, D. Henke, A. Oevermann, V. Jagannathan, J. R. Mickelson, C. Drögemüller

**Affiliations:** 1 Department of Veterinary and Biomedical Sciences, University of Minnesota, Saint Paul, MN 55108 USA; 2 Institute of Genetics, University of Bern, Bern, 3001 Switzerland; 3 Department of Clinical Sciences of Companion Animals, Utrecht University, Utrecht, 3508 CM The Netherlands; 4 Centre for Clinical Veterinary Medicine, Ludwig-Maximilians-University, Munich, 80539 Germany; 5 Department of Small Animal Medicine, University of Leipzig, Leipzig, 04103 Germany; 6 Department of Basic Medical Sciences, Purdue University, West Lafayette, IN 47907 USA; 7 Small Animal Medicine and Surgery, University of Georgia, Athens, GA 30602 USA; 8 Veterinary Sciences Centre, University College Dublin, Dublin, D04 V1W8 Ireland; 9 Department of Pathology, University of California, La Jolla, CA 92093 USA; 10 Department of Population Health and Reproduction, University of California-Davis, Davis, CA 95616 USA; 11 Pathobiology and Population Sciences, The Royal Veterinary College, North Mymms, AL9 7TA UK; 12 Division of Clinical Neurology, University of Bern, Bern, 3001 Switzerland; 13 Division of Neurological Sciences, University of Bern, Bern, 3001 Switzerland

## Abstract

Canine leukoencephalomyelopathy (LEMP) is a juvenile-onset neurodegenerative disorder of the CNS white matter currently described in Rottweiler and Leonberger dogs. Genome-wide association study (GWAS) allowed us to map LEMP in a Leonberger cohort to dog chromosome 18. Subsequent whole genome re-sequencing of a Leonberger case enabled the identification of a single private homozygous non-synonymous missense variant located in the highly conserved metallo-beta-lactamase domain of the *N-acyl phosphatidylethanolamine phospholipase D* (*NAPEPLD*) gene, encoding an enzyme of the endocannabinoid system. We then sequenced this gene in LEMP-affected Rottweilers and identified a different frameshift variant, which is predicted to replace the C-terminal metallo-beta-lactamase domain of the wild type protein. Haplotype analysis of SNP array genotypes revealed that the frameshift variant was present in diverse haplotypes in Rottweilers, and also in Great Danes, indicating an old origin of this second *NAPEPLD* variant. The identification of different *NAPEPLD* variants in dog breeds affected by leukoencephalopathies with heterogeneous pathological features, implicates the NAPEPLD enzyme as important in myelin homeostasis, and suggests a novel candidate gene for myelination disorders in people.

## Introduction

The classification of human leukoencephalopathies was initially based upon pathology and biochemistry and has been applied to disorders caused by toxic, acquired vascular, or infectious insults, as well as inherited disorders^1^. This scheme has recently been updated to a case definition of leukodystrophies that refer to 30 distinct disorders with wasting (dystrophy) of the brain’s white matter (leuko) and a consensus definition of heritable white matter disorders based on neuroimaging^1,2^. Interestingly, nearly half of all patients whose neuroimaging studies indicate white matter disease and whose clinical manifestations suggest a genetic etiology do not receive a specific diagnosis^3^. More than 60 distinct types of genetic leukoencephalopathies (gLE), a recently introduced broader term^1^, are associated with white matter lesions in the central nervous system (CNS), and in people these represent a heterogeneous group of disorders with both highly variable clinical and pathologic manifestations^1,4^. A recent genetic screening of 118 leukoencephalopathy-related genes in 49 patients diagnosed with gLE showed evidence for pathogenic variants in 40.8% of them^5^.

In humans, primary myelin disorders of CNS (so called white matter diseases) are caused by defects in myelin formation and/or maintenance and include dysmyelinating (abnormally formed myelin) diseases, hypomyelinating disorders (decreased myelin production), and spongy vacuolar degeneration of myelin^4^. Myelin disorders have also been reported in miscellaneous domestic animal species including various breeds of dog^6,7^. Although infrequently seen, during the last 40 years several breed-specific forms of myelopathy in which there is lysis of the white matter have been described and termed leukoencephalomyelopathies (OMIA 001788-9615) in Afghan Hounds^8^, Rottweilers^9–12^ and Leonbergers^13^, or as necrotizing myelopathy in Kooiker dogs^14^. Affected dogs present clinically weak and ataxic with loss of conscious proprioception (Supplementary Video S1). Usually these diseases occur in young animals suggesting a hereditary basis.

As similar myelin disorders are known in people, this study aimed to identify the genetic cause of canine leukoencephalomyelopathy (LEMP) in Leonbergers and Rottweilers as complementary models. Herein we report the identification of a causative gene for both these forms of canine LEMP that represents a novel candidate gene for human myelin disorders such as gLE disease.

## Results

### Leukoencephalomyelopathy (LEMP) in Leonbergers is associated with the region of *N-acyl phosphatidylethanolamine phospholipase D* (*NAPEPLD*) on chromosome 18

**Figure 1:**
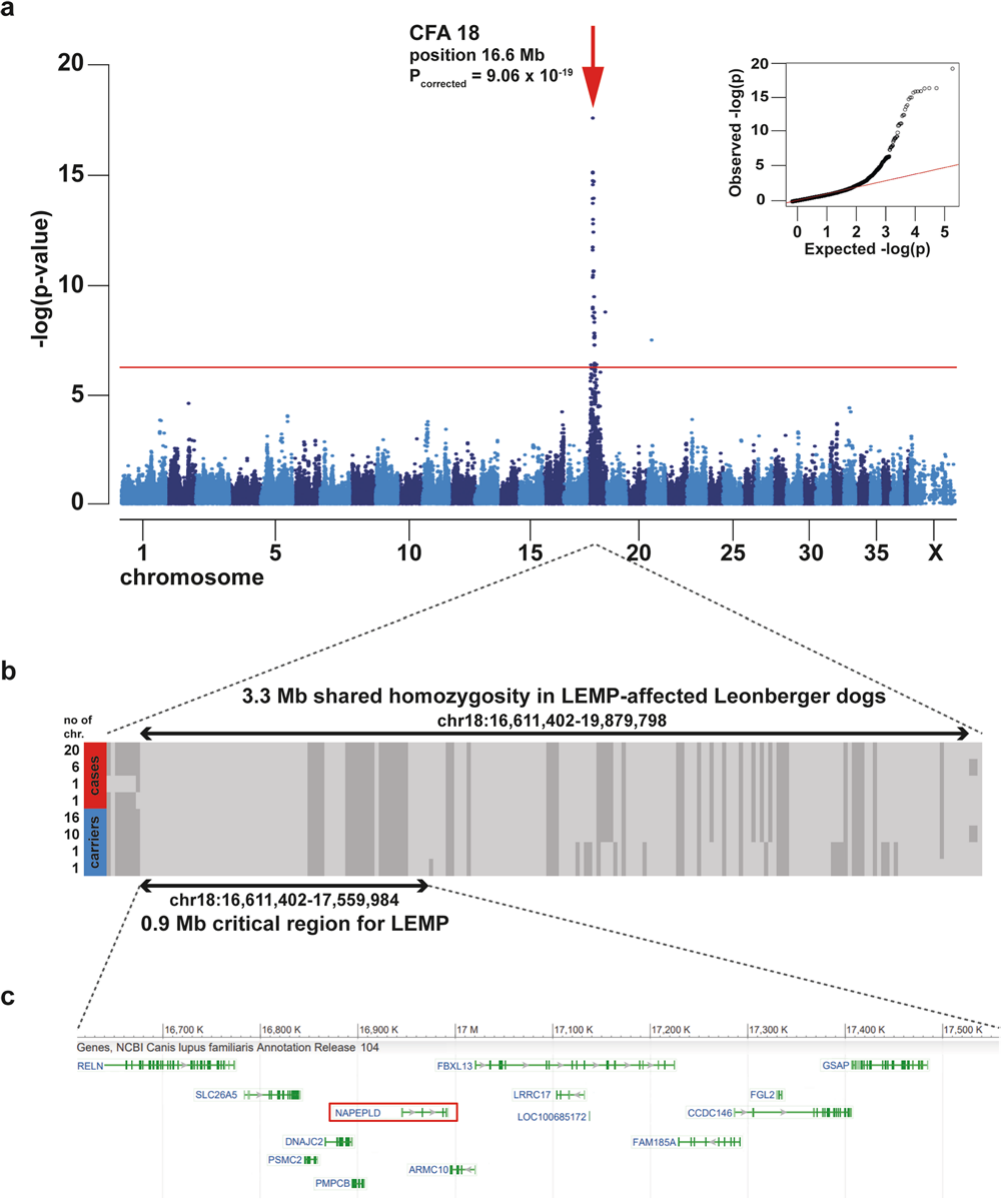
Positional cloning of the LEMP-associated locus in Leonbergers. (**a**) Manhattan plot for the GWAS using 14 LEMP-affected dogs and 186 control dogs is shown and indicates a signal with multiple associated SNPs on chromosome 18. The -log P-values for each SNP are plotted on the y-axis versus each canine autosome and the X chromosome on the x-axis. The red line represents the Bonferroni corrected significance threshold (−log (P) = 6.35). A mixed model analysis corrected for population stratification was carried out as described in the Methods. Inset: Corrected QQ-plot confirms that the actually observed P-values of the best associated markers have stronger association with the trait than expected by chance (null hypothesis, red line). (**b**) Haplotype analysis of SNP array genotypes of 14 cases and 28 carriers allowed fine mapping of the critical region for LEMP to a 0.9 Mb interval. Each line represents a unique haplotype. (**c**) The LEMP-associated region contains 14 loci including *NAPEPLD* gene.

For the GWAS we utilized 14 Leonberger neurological cases clinically compatible with LEMP (ages of onset 1.3–4 years), in which seven cases were confirmed by necropsy, and one dog also confirmed via magnetic resonance imaging (Supplementary Table S1). Additionally, we included 186 neurologically healthy Leonberger controls (eight years and older) based on prior 170k SNP array genotyping data (Fig. 1a). A genomic inflation factor (lambda) of 2.29 indicated the presence of population stratification and possible cryptic relatedness. We performed a multidimensional scaling analysis revealing no indication for clustering of cases outside the controls (Supplementary Fig. S1). We therefore performed an association analysis using the mixed model function that resulted in lambda dropping to 1.187. We obtained a highly significant association signal on chromosome 18 (Fig. 1a; p_corrected_ = 9.06 × 10^−19^). Haplotype analysis of the LEMP-affected Leonbergers showed a 3.3 Mb area of extended shared homozygosity from positions 16.6 to 19.9 Mb (Fig. 1b).

### A missense variant in the *NAPEPLD* gene is associated with LEMP in Leonbergers

**Figure 2:**
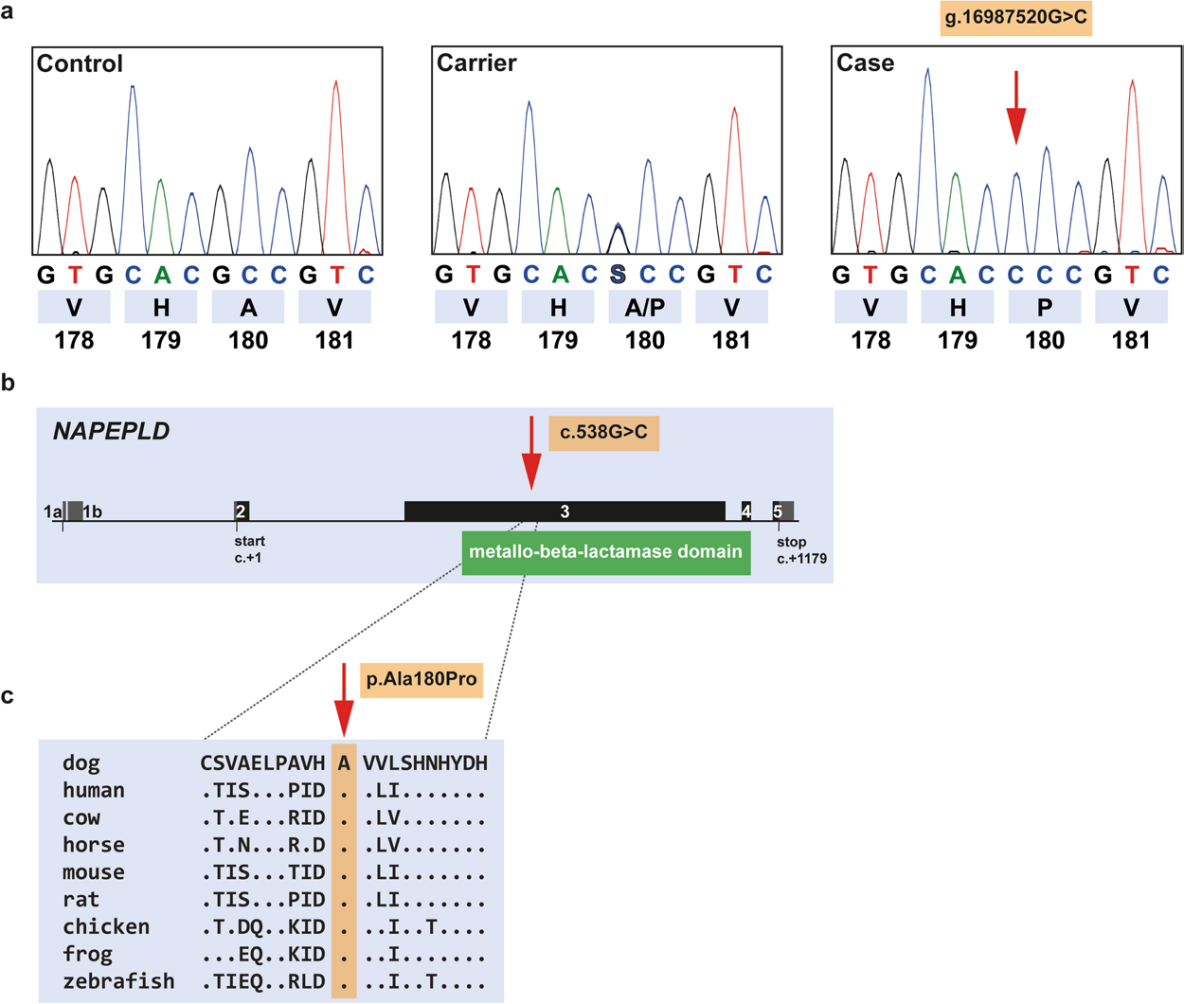
The *NAPEPLD* missense variant detected in LEMP-affected Leonbergers. (**a**) Chromatograms of wild type, carrier, and an affected dog indicate the c.538 G > C variant which changes codon 180 (shown below). (**b**) The variant is located in exon 3 of canine *NAPEPLD* that encodes a functionally important domain of the NAPEPLD protein. (**c**) The predicted p.Ala180Pro exchange affects an evolutionary conserved residue. The multiple sequence amino acid alignment was done using accessions XP_005631036.1 (*Canis lupus familiaris*), NP_001116310.1 (*Homo sapiens*), NP_001015680.1 (*Bos taurus*), XP_014594420.1 (*Equus caballus*), NP_848843.1 (*Mus musculus*), NP_955413.1 (*Rattus norwegicus*), NP_001025901.1 (*Gallus gallus*), XP_002933136.1 (*Xenopus tropicalis*) and NP_001074082.2 (*Danio rerio*).

Whole genome re-sequencing (WGS) was performed on a Leonberger LEMP case homozygous for the associated haplotype. Subsequently, sequence variants in the mapped interval were called. The pedigree analysis (Supplementary Fig. S2) and the large size of the homozygous interval indicated a relatively young origin of the variant and a likely recessive mode of inheritance. Thus, we assumed that the causative variant should be absent from breeds unrelated to the Leonbergers. A total of 32 variants in the interval unique to the sequenced case remained after filtering against 201 control genomes of 66 different dog breeds and three wolves (Supplementary Table S2). Only a single variant (chr18:g.16987520 G > C) was predicted to affect the coding sequence of an annotated gene (Supplementary Table S3). Sanger sequencing confirmed the presence of this variant (Fig. 2a) and its nearly perfect association to the LEMP phenotype (Supplementary Fig. S2). This private non-synonymous variant in the *N-acyl phosphatidylethanolamine phospholipase D* (*NAPEPLD*) gene (c.538 G > C) is located within the metallo-beta-lactamase domain of the encoded protein (Fig. 2b). It is predicted to alter the sequence of codon 180 resulting in the replacement of alanine by proline (p.Ala180Pro). Multiple species amino acid sequence alignment showed that the wild type residue at the affected position is conserved across NAPEPLD orthologues in vertebrates including the zebrafish (Fig. 2c). Software-based analysis of the NAPEPLD amino acid exchange characterized the variant as probably damaging (PolyPhen 2), deleterious (SIFT), pathogenic (MutPred2) or disease causing (Mutation Taster). An mRNA-seq experiment on a spinal cord sample of a LEMP-affected Leonberger was carried out and revealed no evidence for alternative splicing of *NAPEPLD* in comparison to a spinal cord sample from a control dog (not shown).

To further investigate the frequency of the *NAPEPLD* variant in Leonbergers, and its segregation with disease, we genotyped in total 7,086 dogs (Table 1). This includes all 200 dogs from the GWAS, two previously described cases^13^, 11 additional LEMP-affected Leonbergers, as well as 574 dogs with owner reported health updates indicating no neurological problems within the first eight years of life. There was a highly significant difference in *NAPEPLD* allele frequencies between LEMP cases and these controls using a standard chi-square test (p < 1.5 × 10^−89^). The absence of affected heterozygotes in this large cohort supports a recessive mode of inheritance (standard chi-square test p < 7.9 × 10^−108^). However, approximately 1% (6/574) of non-affected dogs at eight years of age were homozygous for the mutant allele, indicating reduced penetrance (Table 1

**Table 1:** *NAPEPLD* c.538 G > C genotype frequencies in Leonbergers and 7 other related breeds.

Breed	Status	Total	G/G (homozygous normal; N/N)	C/G (heterozygous; D/N)	C/C (homozygous mutant; D/D)
Leonberger		7,086	5,956	1,054^*^	76^1^
	Affected^2^	27			27^1^
	Non-affected^#,2^	574	486	82	6
	Unknown^§^	6,485	5,470	972	43
Great Danes^§^		262	262		
St. Bernard^§^		47	47		
Newfoundland^§^		10	10		
Entlebucher Mountain dog^§^		10	10		
Appenzeller Mountain dog^§^		8	8		
Bernese Mountain dog^§^		7	7		
Greater Swiss Mountain dog^§^		6	6		

^*^Including 10 obligate carriers (dam/sire of LEMP cases).

^#^Owner reported no signs of LEMP in dogs older than 8 years.

^§^Dogs without known phenotype status that were submitted for diagnostic purposes.

^1^Includes the two cases reported before^13^.

^2^Allele frequency difference p < 1.5 × 10^−89^.

). Furthermore, we genotyped nearly 6,500, mostly young, Leonbergers with unknown phenotype status, that were submitted for diagnostic purposes, which well represents the global population of the breed. The genotype frequency of homozygous mutant dogs in this cohort was 0.6%. Altogether, the mutant allele frequency in the global population of 7,086 Leonbergers was estimated as 8.5%.

Finally, the analysis of 28 heterozygous dogs carrying the LEMP-associated *NAPEPLD* allele identified shorter versions of the variant-containing haplotype due to recombination events. This enabled narrowing of the shared region surrounding the *NAPEPLD* allele on chromosome 18 to approximately 0.9 Mb (bp position 16,611,402 to 17,559,984; Fig. 1b). This shared haplotype contains 13 annotated protein-encoding genes and one pseudogene (Fig. 1c).

### A frameshift variant in the *NAPEPLD* gene is associated with LEMP in Rottweilers and also occurs in Great Danes

**Figure 3:**
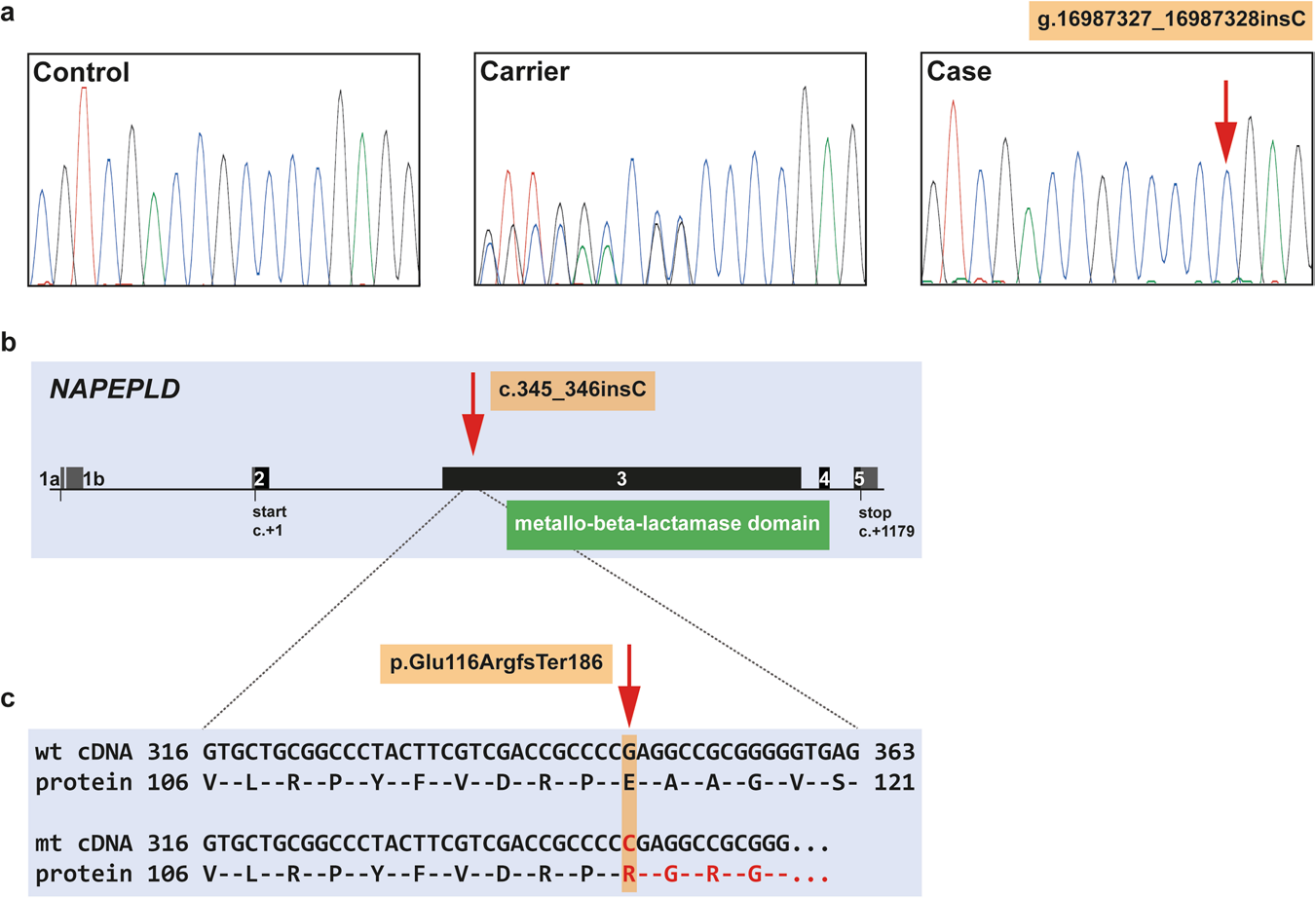
The *NAPEPLD* frameshift variant detected in LEMP-affected Rottweilers. (**a**) Chromatograms of wild type, carrier, and an affected dog indicate the c.345_346insC variant. (**b**,**c**) The schematic representation of the canine *NAPEPLD* gene indicates that the 1 bp insertion is located in exon 3 and leads to a frameshift which is predicted to produce a novel 186 amino acid long C-terminus of NAPEPLD and replaces the metallo-beta-lactamase domain of the wild type protein.

We utilized a total of four LEMP-affected Rottweilers to evaluate whether the *NAPEPLD* gene harbored possible disease-causing variants in this breed. This cohort includes the two previously described cases^11,12^ and two additional dogs (Supplementary Table S4). All four coding exons of the *NAPEPLD* gene were Sanger sequenced in the LEMP-affected Rottweilers. All were clear of the Leonberger missense variant, but a frameshift variant in exon 3 (c.345_346insC) was discovered in which the four LEMP-affected Rottweilers were homozygous (Fig. 3a). This 1 bp insertion is predicted to replace the C-terminal metallo-beta-lactamase domain of the wild type protein by a recoded peptide of 186 amino acids (p.Glu116ArgfsTer186) without any sequence similarity (Fig. 3b,c). The *NAPEPLD* frameshift allele (chr18: g.16987327_16987328insC) frequency in a population of 229 non-affected Rottweilers was 3.7% with no homozygous mutant dogs observed (Table 2). To confirm the focus on the *NAPELD* gene we performed a GWAS with 3 affected Rottweilers with passing SNP genotyping call rate and identified the same chromosome 18 locus (p_corrected_ = 7.06 × 10^−15^) as found in Leonbergers, that spanned the *NAPEPLD* gene (Supplementary Fig. S3). Further, WGS of an LEMP-affected Rottweiler confirmed the 1 bp insertion which was identified via Sanger sequencing and showed no further variants in the *NAPEPLD* gene. We also screened related breeds for the presence of this frameshift variant and identified non-affected heterozygous dogs in Great Danes, and an allele frequency of 4.5% in this breed cohort (Table 2). This variant was not present among the 201 sequenced control genomes of 66 different dog breeds and three wolves (Supplementary Table S2).

**Table 2:** *NAPEPLD* c.345_346insC genotype frequencies in Rottweilers, Great Danes and 6 other related breeds.

Breed	Status	Total	wt/wt (homozygous normal; N/N)	ins/wt (heterozygous; D/N)	ins/ins (homozygous mutant; D/D)
Rottweiler		233	212	17	4^1^
	Affected^2^	4			4^1^
	Non-affected^2^	72	64	8	
	Unknown^§^	157	148	9	
Great Danes^§^		262	238	24	
St. Bernard^§^		47	47		
Newfoundland^§^		10	10		
Entlebucher Mountain dog^§^		10	10		
Appenzeller Mountain dog^§^		8	8		
Bernese Mountain dog^§^		7	7		
Greater Swiss Mountain dog^§^		6	6		

^§^Dogs without known phenotype status that were submitted for diagnostic purposes.

^1^Includes the two cases reported before^11,12^.

^2^Allele frequency difference p < 2.4 × 10^−17^.

**Figure 4:**
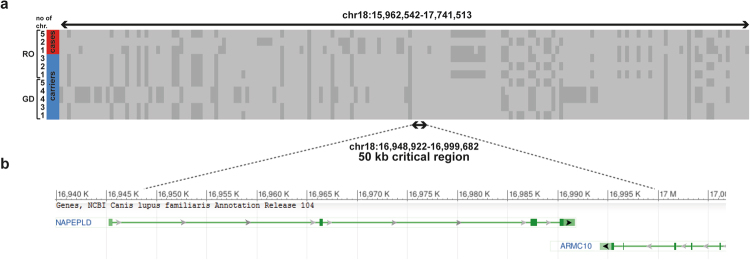
Across breed haplotype analysis in Rottweilers and Great Danes indicates an old mutation event. (**a**) Diverse haplotypes were detected exploring SNP array genotypes of four LEMP-affected Rottweilers (RO) and 23 heterozygous carriers of the *NAPEPLD* frameshift variant in Rottweilers and Great Danes (GD). Each line represents a unique haplotype. (**b**) A 50 kb-sized identical haplotype in all dogs contains segments of the canine *NAPEPLD* and *ARMC10* genes.

Subsequent haplotype analysis of 170k SNP array genotypes revealed that the *NAPEPLD* frameshift variant was present on three diverse haplotypes in affected Rottweilers (Fig. 4a). We then genotyped additional heterozygous Rottweilers and Great Danes to study the haplotype diversity in a 1.5 Mb interval surrounding the *NAPEPLD* gene. This revealed a collection of extended haplotypes associated with the frameshift variant (Fig. 4a). Nonetheless, an identical 50 kb sub-haplotype containing segments of the canine *NAPEPLD* and *ARMC10* genes was identified in all homozygous and heterozygous dogs (Fig. 4b).

### Variable histopathological phenotype in *NAPEPLD* homozygous dogs within and across breeds

**Figure 5:**
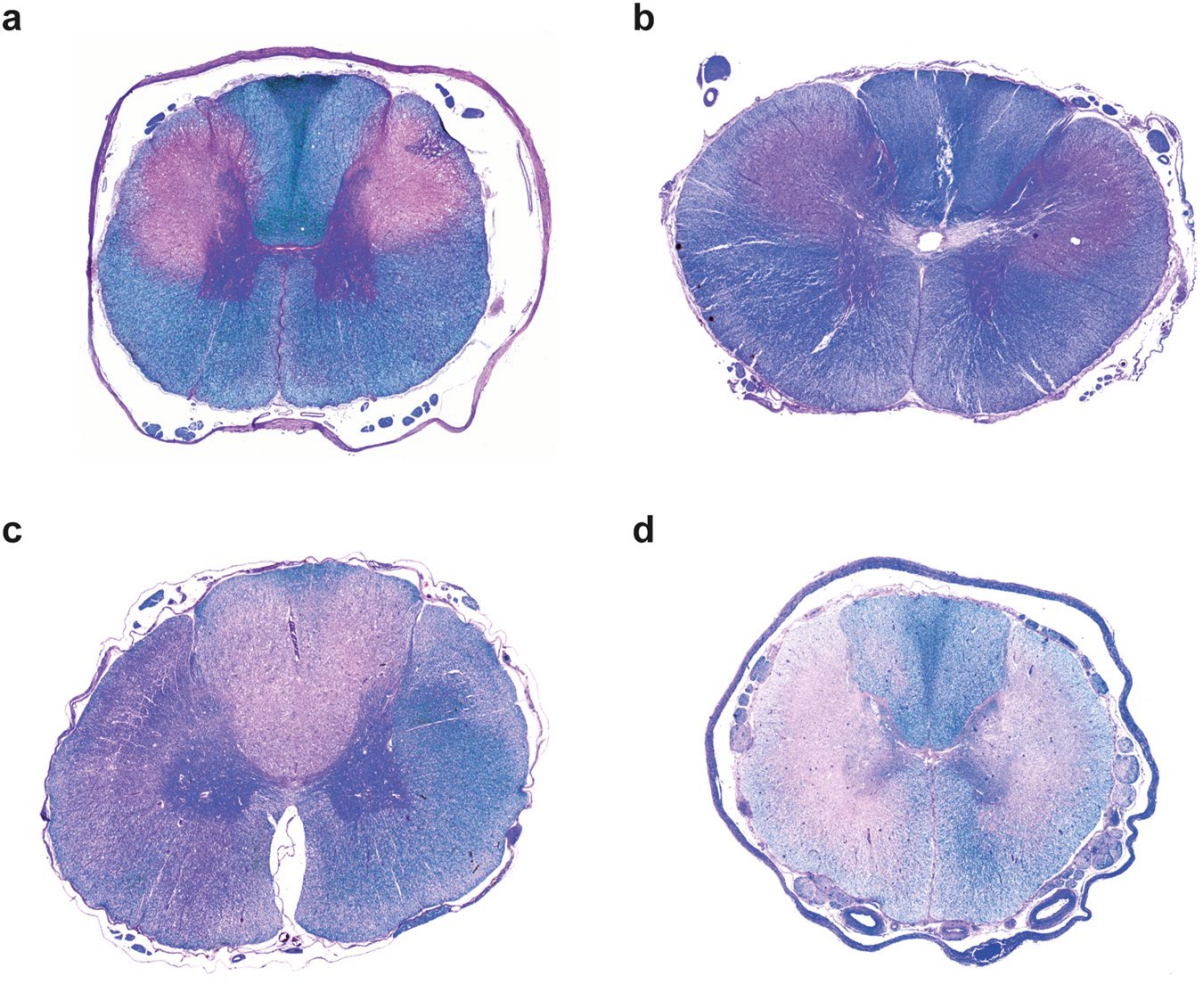
Phenotypic variability of transverse spinal cord sections of LEMP-affected dogs. Combined luxol fast blue/hematoxylin & eosin stain of paraffin sections. (**a**) Thoracic spinal cord of a Leonberger case L1 with typical LEMP lesions as previously described^13^. Note the bilateral-symmetrical loss of myelin in the corticospinal tracts as indicated by the loss of the blue color. (**b**) Cervical spinal cord of a previously described^12^ Rottweiler case R1 with similar lesions as in (**a**). This dog had severe white matter loss in some areas combined with axonal loss, infiltration by macrophages and capillary hypertrophy. (**c**) Thoracic spinal cord of Leonberger case L3. The quality of lesions is similar as in (**a**), but the distribution is different with lesions being most severe in the dorsal tracts. (**d**) Thoracic spinal cord of Rottweiler case R3 showing similar lesions, which are less defined and more widespread encroaching into lateroventral tracts.

**Figure 6:**
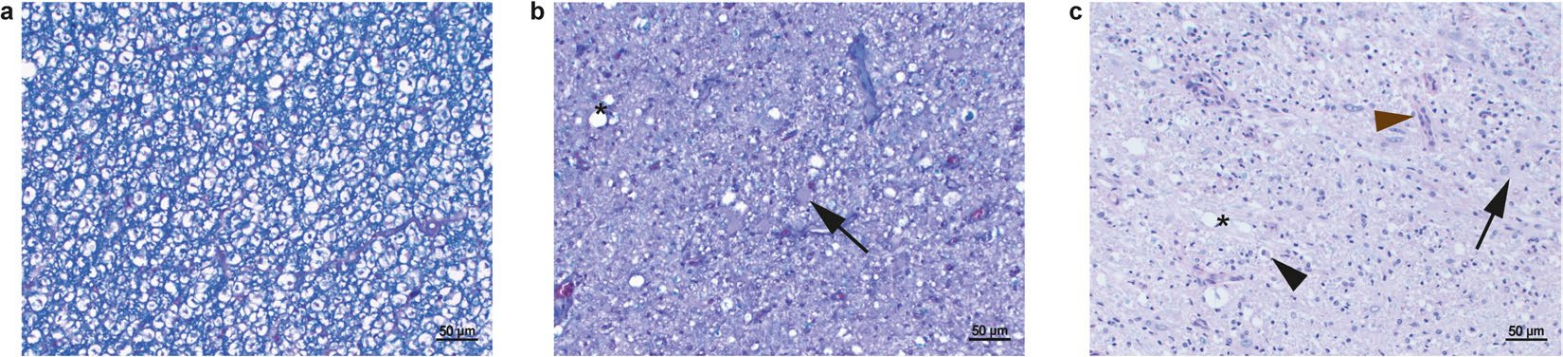
Histopathology of the spinal cord of LEMP-affected dogs. Combined luxol fast blue/hematoxylin & eosin stain of paraffin sections. (**a**,**b**) Spinal cord sections of previously described^11^ Rottweiler case R2 showing deep blue staining of the normal myelin in an unaffected area (**a**) and an affected spinal cord area (**b**) exhibiting loss of blue myelin staining, vacuoles (asterisk), and large gemistocytic astrocytes (arrow). (**c**) Affected spinal cord area of the previously described Rottweiler case R1^12^ exhibiting severe loss of blue myelin staining, vacuoles (asterisk), infiltration by macrophages (black arrowhead), capillary hypertrophy (brown arrowhead) and large gemistocytic astrocytes (arrow).

After variant identification we histopathologically re-evaluated eight neurologically affected Leonbergers genotyped as homozygous mutant for the c.538 G > C variant, including the two previously described cases^13^, and three neurologically affected Rottweilers genotyped as homozygous mutant for the c.345_346insC variant, including two reported LEMP-affected dogs from Germany^11^ and the US^12^. Stained tissue sections available for re-evaluation varied between dogs, however clear variation in histological lesions was observed. Nine out of these eleven dogs had histopathological lesions compatible with LEMP (Leonberger cases L1-6 (Supplementary Table S1), Rottweiler cases R1-3 (Supplementary Table S4)). In all nine dogs the spinal cord was affected. Additionally, in four LEMP-affected dogs, of which the brain was also available, a specific pattern with lesions involving the spinal tract of the trigeminus, cerebellar peduncles, cerebellar medulla, pyramids, crus cerebri, and optic tract was observed. No lesions were present in the spinal nerve roots. Generally, the nine dogs compatible with LEMP suffered marked loss of myelin (Fig. 5). The myelin was replaced by numerous fibrillary and gemistocytic astrocytes (Fig. 6). Within and around the gliotic area scattered dilated myelin sheaths containing gitter cells were present, and a few scattered swollen axons were observed. Minimal Wallerian-like degeneration was observed in areas distant from the areas of myelin loss. Lesions were predominantly bilaterally symmetrical (Fig. 5), although in some areas a marked asymmetry could be observed.

There was variation in severity and topography between cases. Three cases (L1, L2, and R2) exhibited lesions predominantly affecting the lateral corticospinal tract and encroaching on the dorsal spinocerebellar, rubrospinal and the lateral spinothalamic tracts as previously described. In three dogs (R1, L5, and L6) lesions were similar in distribution, but very severe, being characterized by severe loss of white matter with infiltration of macrophages and capillary prominence. In three other cases (R3, L3 and L4) the distribution was different with lesions being most severe in the dorsal funiculi or being more widespread. In most dogs, axonal changes were relatively mild and confined to the areas of severe myelin loss indicating a primary myelin disorder. In contrast, one Rottweiler case (R3) with severe white matter lesions exhibited more conspicuous axonal degeneration, and Bielschowsky stain revealed reduced axonal density in these areas.

Interestingly, two Leonbergers exhibited histopathological lesions not compatible with LEMP. One Leonberger case (L8) suffered axonal degeneration in the peripheral nerves associated with denervation atrophy of the skeletal muscle (Supplementary Fig. S4) compatible with Leonberger polyneuropathy^15^. Additionally, this dog exhibited scattered axonal degeneration in the spinal cord without conspicuous myelin loss (Supplementary Fig. S4). The second deviating Leonberger case (L7) had no lesions in the examined spinal cord section, but a well-defined, plaque-like area of demyelination and gliosis in the corona radiata (Supplementary Fig. S5).

## Discussion

We investigated a possible genetic basis for leukoencephalomyelopathy (LEMP) in Leonbergers and Rottweilers. The pathological lesions in both breeds were reported clearly to be demyelinating but the distribution, restricted to the white matter of brain and spinal cord, remained unclear^11,13^. Our studies revealed two independent non-synonymous variants affecting the canine *NAPEPLD* gene, a gene with no known role in myelination or myelinogenesis of oligodendrocytes.

The endocannabinoid system consists of endocannabinoids, cannabinoid receptors and enzymes, such as NAPEPLD, involved in the synthesis and degradation of endogenous ligands^16^. It is a widespread neuromodulatory system which plays important roles in central nervous system development, synaptic plasticity, and the response to endogenous and environmental insults^16^. Some endocannabinoids are supposed to have a neuroprotective function^17^. The NAPEPLD protein is a membrane-bound phospholipase D type enzyme that catalyzes the release of N-acylethanolamine from N-acyl-phosphatidylethanolamine^18^, and in so doing generates N-arachidonoylethanolamine, a ligand of cannabinoid and vanilloid receptors^19^. NAPEPLD is the enzyme catalyzing the production of arachidonoylethanolamine in animal tissues, and is structurally different from other known phospholipases D^19^. Nonetheless, it belongs to the zinc metallohydrolase family of the beta-lactamase fold, which is involved in a variety of biological events including antibiotic resistance, DNA repair and RNA maturation^20^. Several single point mutants, affecting highly conserved histidine and aspartic acid residues in the metallo-beta-lactamase domain involved in the binding to Zn^2+^, are known to be catalytically inactive or have reduced enzyme activity^20,21^. The NAPEPLD protein forms a homodimer composed of two interconnected subunits, partly separated by an internal channel, and uniquely adapted to associate with phospholipids^22^. Recently, it was found that binding of bile acid enhances dimer assembly, and stimulates the NAPEPLD enzyme to favor the selective production of the endocannabinoid anandamide and other fatty acid ethanolamides^23^.

Mice with a targeted disruption of the *NAPEPLD* gene were viable and healthy, but displayed reductions in very long-chain saturated and mono-unsaturated N-acyl ethanolamines in the central nervous system^24^. Brain tissue from mice lacking *GDE1* and *NAPEPLD* showed a near-complete loss in N-acyl-phosphatidylethanolamine conversion to N-acyl ethanolamines, but bulk brain levels of N-acyl ethanolamines were unaltered^25^. It was concluded that, both GDE1 and NAPEPLD make partial contributions to the biosynthesis of anandamide and other N-acyl ethanolamines *in vivo*^25^. No brain pathology was investigated in these knock-out mice. Pharmacological administration of agonists and antagonists of cannabinoid receptors in rats showed that the activation of both receptors is needed to augment the expression of myelin basic protein in the subcortical white matter^26^. Although most studies have focused on the role of endocannabinoids in neuronal differentiation, essential data suggest that these signalling lipids are also important in myelination of long-range axons to increase their conductance velocity^27^. Therefore we speculate that disruption in the production of endocannabinoids by a mutation in the *NAPELPD* gene could be a mechanism of demyelination. Recently, a marked upregulation of type-2 cannabinoid receptors in the spinal cord of dogs showing *SOD1*-associated degenerative myelopathy was reported, supporting the assumed neuroprotective function of the endocannabinoid system in neurodegenerative disorders^28^. For the first time, our study implicates a significant role of the NAPEPLD enzyme, and thereby the endocannabinoid system, in maintaining the white matter. In LEMP-affected dogs it was previously speculated that the primary defect might be located in neurons or axons^13^. Therefore the NAPEPLD enzyme could be involved in the axon-myelin crosstalk and might improve the understanding of the currently poorly characterized molecular and cellular mechanisms that impact the differentiation of oligodendrocytes and myelination^29^. Furthermore, there is as yet no association known for involvement of the *NAPEPLD* gene in myelin disorders in people^19^. The group of heterogeneous leukoencephalopathies, characterized by white matter abnormalities affecting the CNS, is associated with various genes; still, the genetic cause in about every second patient is unknown^30,31^. We suggest the *NAPEPLD* gene is worthy of investigation as a possible molecular basis of genetic leukoencephalopathies.

There are 173 observed human *NAPEPLD* coding region variants depicted in Exac Browser^32^: 43 are synonymous, 123 are missense, and 4 are loss of function; with 3 CNVs. The synonymous, missense and CNV variants all have positive Z scores, indicating increased intolerance to variation and fewer variants in the gene than expected. The probability of loss of function intolerance is pLI = 0.06, which places *NAPEPLD* gene variants as most likely being recessive, where heterozygous loss of function variants are often tolerated^32^. One missense human SNP (rs367936558) alters the same position of the human NAPEPLD protein as the variant found in Leonbergers; however the alanine residue is exchanged by valine (p.Ala180Val), not by proline as found here. This human variant is present in only 1 out of 60,704 patients, and in heterozygous state (allele frequency of 8.237e^−06^)^32^.

The two disease-associated canine *NAPEPLD* variants affect the highly conserved metallo-beta-lactamase domain: changing a single amino acid in LEMP-affected Leonbergers, and removing the entire domain by a frameshift in Rottweilers. Interestingly, with the exception of two dogs, the general disease phenotype in these two breeds is very similar, although we have noticed a certain variability comparing dogs within the affected breeds sharing the identical homozygous mutant genotype. This could be explained either by disease progression over time, or by the individual genetic background and epistatic effects to unknown variation in possible modifier genes^33^. Recently it was shown that variations in SP110-mediated gene transcription may underlie, at least in part, the variability in risk for developing canine degenerative myelopathy among Pembroke Welsh Corgis that are homozygous for the disease-related *SOD1* mutation^34^. Similar efforts might be carried out to identify modifier genes explaining the observed variability in developing LEMP in Leonbergers. In addition, by genotyping several thousand Leonbergers, we noticed about 1% of dogs harboring the homozygous mutant genotype with no reported clinical manifestation (Table 1). This indicates a reduced penetrance of the *NAPEPLD* missense variant and supports recent findings in human genetics indicating that incomplete penetrance for presumed Mendelian diseases is likely more common than previously believed^35^.

Furthermore, the neuropathological examination of eight *NAPEPLD* homozygous mutant Leonbergers indicated that one dog had a primary axonal disorder, not a myelin disorder, which was first noticed clinically as late as 7.5 years of age (Leonberger case L8, Supplementary Table S1). As we have learned recently in polyneuropathy-affected Leonbergers, where only approximately every third polyneuropathy-diagnosed Leonberger can be explained by the reported *ARHGEF10* or *GJA9* variants^36,37^, this case could possibly be explained by an independent mutation in an unknown gene causing polyneuropathy, although a possible effect of the *NAPEPLD* genotype could not be ruled out. Altogether, this nicely highlights the limits to precisely clinically diagnosing neurological diseases in dogs. Furthermore, we noticed in Leonbergers that some dogs were initially diagnosed as polyneuropathy-affected, although in fact they were suffering from LEMP. Disease awareness has to be taken into account as well, when dogs that have not been diagnosed by a board-certified neurologist are used for genetic studies. Finally, variation in histopathological phenotypes and genotype-phenotype correlations in the population may also be influenced by the fact that the samples available were examined retrospectively from dogs of varying age and geographic locations.

In the Rottweilers it is unclear whether the truncated NAPEPLD protein produced by the frameshift variant, with more than 70% of the normal protein missing, is actually expressed. Furthermore, it is very unlikely that the predicted mutant protein with a recoded peptide of 186 amino acids before the newly encoded stop codon, without any sequence similarity to the normal protein (Supplementary Fig. S6), would fulfill any physiological function. This mutant protein would also have the functionally important metallo-beta-lactamase domain missing (Fig. 3). It is therefore more likely that the mutant mRNA is targeted by non-sense-mediated decay, thus the deleterious canine *NAPEPLD* variant represents the most likely causative variant in the four LEMP-affected Rottweilers.

Our results provide strong evidence for allelic heterogeneity in canine LEMP, where two independent *NAPEPLD* variants were found in Leonbergers and Rottweilers. In addition, we found several Great Danes being heterozygous carriers of the variant identified in Rottweilers, but so far we have not seen a homozygous mutant dog in this third breed. Although all three breeds are part of the Molossian section of the World Canine Organization classification, Leonbergers belong to the Mountain type dogs, and both Rottweilers and Great Danes represent the group of Mastiff type dogs. According to a recent study on the development of modern dog breeds all three breeds belong to separate clades, but show significant haplotype sharing^38^. This example of a similar canine disease occurring in different breeds caused by independent variants affecting the same gene, is comparable to what has been shown before, for example, in canine *NDRG1*-related polyneuropathy (OMIA 002120-9615)^39,40^. The missense variant detected in Leonbergers is probably caused by a recent mutation event as the associated haplotype encompasses a 0.9-Mb-sized region. On the other hand, the frameshift variant detected in LEMP-affected Rottweilers and in Great Dane carriers most likely has a quite old origin, as it exists on a relatively small-sized common haplotype of 50 kb.

In conclusion, here we report the identification of two *NAPEPLD*-associated variants in LEMP-affected dogs. Our results indicate a recessive mode of inheritance in each, albeit with a slightly reduced penetrance, and enable the development of genetic tests for veterinary diagnostic and breeding purposes. Our study describes a canine neurological disease with distinctive pathological features and implicates the NAPEPLD protein as an important enzyme in myelin homeostasis. Finally, our results reveal a novel candidate gene for myelin disorders such as the genetic leukoencephalopathies in humans.

## Methods

### Animals

Written consent was obtained from all dogs’ owners. Dog samples were obtained primarily via elective owner submission for diagnostic purposes or were submitted for genotyping of the previously reported polyneuropathy-associated variants in Leonbergers. Most of these dogs do not have complete medical information and were used only for a population study. Blood collection from dogs does not require anesthesia and the study was approved according to the national guidelines for animal welfare by the Institutional Animal Care and Use Committees (IACUC) of the University of Minnesota, and by the Cantonal Committee for Animal Experiments (Canton of Bern; permits 23/10, 48/13 and 75/16) for the University of Bern.

All invasive procedures were performed *post-mortem* either on animals that had died of natural causes, or after euthanasia, thus no ethical evaluation was required. All methods were performed in accordance with the relevant guidelines and regulations of the University of Minnesota and the University of Bern.

Genomic DNA was isolated from blood using either the Gentra PureGene blood kit (Qiagen) or the Maxwell RSC whole blood DNA kit (Promega). The phenotypic characterization of LEMP in Leonbergers and Rottweilers has been described elsewhere and the previously established criteria to select cases and controls were applied^36^. Samples from a total of 7,086 Leonbergers, including 213 dogs with detailed phenotype records (Supplementary Table S1), and 233 Rottweilers, including four dogs with detailed phenotype records (Supplementary Table S4) were used during this study. All Leonbergers were genotyped for the polyneuropathy-associated *ARHGEF10* and *GJA9* variants as previously described^36,37^ to test for underlying neurological disease. Furthermore, DNA samples of 262 Great Danes, 47 St. Bernards, 10 Newfoundlands, 10 Entlebucher Mountain dogs, 8 Appenzeller Mountain dogs, 7 Bernese Mountain dogs, and 6 Greater Swiss Mountain dogs were taken from the Vetsuisse Biobank.

### Histopathology

Histopathological samples of eight Leonbergers and four Rottweilers were examined. Two to five µm thick sections from 10% neutral-buffered formalin fixed and paraffin-embedded brain and spinal cord were retrieved from histopathological archives of different diagnostic labs. These included combined luxol fast blue/hematoxylin and eosin stained sections or combined luxol fast blue/periodic acid Schiff stained sections. In five cases, Bielschowsky silver stained sections and in one case sections labeled with PGP 9.5 immunohistochemistry were available for evaluation of axonal density. Tissue sections were examined by light microscopy.

### SNP array genotyping

Genomic DNA samples of 200 Leonbergers, 10 Rottweilers, and 17 Great Danes were genotyped with the Illumina CanineHD BeadChip array by GeneSeek/Neogen for 173,662 SNP markers. In Leonbergers we performed pruning of genotyping data as described previously^36^ and 112,833 SNPs remained for genome-wide association study (GWAS). Fourteen LEMP-affected dogs and 186 controls (i. e. dogs eight years and older that showed no signs of neurological disease) were analyzed with the mixed model from the GenABEL library^41^ and the hglm package^42^ in the R environment that corrects for the population stratification. Multidimensional scaling analysis was carried with the GenABEL^41^. We used 173k SNP data of additional 147 Rottweilers as controls, which were publically available from previous projects^43,44^, to perform GWAS. Due to the limited number of cases in Rottweilers, only a fast score test (GenABEL) for association corrected for possible stratification by principal components was performed. Haplotypes around the significantly associated locus were constructed using fastPHASE^45^. All genome positions refer to the CanFam3.1 reference sequence assembly.

### Whole genome sequencing

We performed a whole-genome sequencing of a LEMP-affected Leonberger. Briefly, we prepared a fragment library with 300 bp insert size and collected ~200 million 2 × 100 bp paired-end reads on a HiSeq. 2000 instrument (Illumina, San Diego, USA), which corresponds to roughly 17x coverage. The reads were mapped against the dog reference genome assembly (CanFam3.1) as described before^36^. The annotation version CanFam3.1.75 (http://www.ensembl.org) was used to predict the functional effects of detected variants as described previously^36^. In addition, whole-genome sequencing of a LEMP-affected Rottweiler (case R2; 13× coverage) was performed accordingly.

The IGV-viewer software^46^ was used for visual inspection of sequence variants to exclude any structural variants in the critical region. For variant filtering we used 204 control genomes, which were either publicly available^47^ or produced during other projects of our group. A list of these control genomes is given in Supplementary Table S2.

### Gene analysis

We used the dog CanFam 3.1 reference genome assembly for all genomic analyses. The chromosome 18 reference sequence has a gap after *NAPEPLD* exon 4 and does not include exon 5 (Supplementary Fig. S7). A partial sequence of intron 3, the entire exon 4, intron 4 and exon 5 are retrievable on the unplaced contig Un_JH373889 of the CanFam 3.1 assembly (Supplementary Fig. S7). Numbering within the canine *NAPEPLD* transcript corresponds to the mRNA sequences within study accession PRJEB22251. The predicted effects of the mutations were evaluated by PolyPhen2^48^, SIFT^49^, Mutation Taster^50^, and MutPred2^51^.

### RNA-seq

We isolated total RNA from spinal cord samples from a single LEMP-affected Leonberger and a Labrador control using the RNeasy Fibrous Tissue Mini kit (Qiagen). Prior to RNA extraction, the tissue was mechanically disrupted using the TissueRuptor device (Qiagen). The RNA samples were transformed into Illumina TruSeq libraries and 2 × 150 bp sequencing reads were obtained on a HiSeq3000 instrument (Illumina). RNA-seq data analysis was done as described before^52^.

### Sanger sequencing

We used Sanger sequencing to confirm the candidate *NAPEPLD* variant c.538 G > C in the affected Leonberger and to amplify the coding exons of the *NAPEPLD* gene in the affected Rottweilers. We amplified PCR products (primers are shown in Supplementary Table S5) using AmpliTaqGold360Mastermix (Life Technologies) and purified PCR products were directly sequenced on an ABI3730 capillary sequencer (Life Technologies). The sequence data were analyzed using Sequencher 5.1 software (GeneCodes).

### Data Availability

Genome sequencing data were deposited in the European Nucleotide Archive (ENA, http://www.ebi.ac.uk/ena): The LEMP-affected Leonberger (sample accession number SAMEA103935360 within study accession PRJEB16012) and the LEMP-affected Rottweiler (sample accession number SAMEA3121337 within study accession PRJEB7735). RNAseq data were deposited in the ENA under sample accession number SAMEA103936001 within study accession PRJEB20118. The mRNA sequences for the canine *N-acyl phosphatidylethanolamine phospholipase D (NAPEPLD)* gene were deposited in the ENA under sample accession number LT906616 and LT906617 within study accession PRJEB22251.

## Electronic supplementary material

Supplementary Information

Supplementary Video
