# Supplementary material for: Canine NAPEPLD-associated models of human myelin disorders
Source: Sci Rep. 2018 Apr 11;8:5818. doi: 10.1038/s41598-018-23938-7 (PMC5895582; doi:10.1038/s41598-018-23938-7)
Supplement: Supplementary file 1 — Supplementary Information [file 41598_2018_23938_MOESM1_ESM.pdf]

Canine *NAPEPLD*-associated models of human myelin disorders

K.M. Minor[1+], A. Letko[2+], D. Becker[2], M. Drögemüller[2], P.J.J. Mandigers[3], S.R. Bellekom[3], P.A.J. Leegwater[3], Q.E.M. Stassen[3], K. Putschbach[4], A. Fischer[4], T. Flegel[5], K. Matiasek[4], K.J. Ekenstedt[6], E. Furrow[1], E.E. Patterson[1], S.R. Platt[7], P.A. Kelly[8], J.P. Cassidy[8], G.D. Shelton[9], K. Lucot[10], D.L. Bannasch[10], H. Martineau[11], C.F. Muir[11], S.L. Priestnall[11], D. Henke[12], A. Oevermann[13], V. Jagannathan[2], J.R. Mickelson[1++], C. Drögemüller[2++*]

[1]Department of Veterinary and Biomedical Sciences, University of Minnesota, Saint Paul, MN, 55108, USA

[2]Institute of Genetics, University of Bern, Bern, 3001, Switzerland

[3]Department of Clinical Sciences of Companion Animals, Utrecht University, Utrecht, 3508 CM, The Netherlands

[4]Section of Clinical & Comparative Neuropathology, Centre for Clinical Veterinary Medicine, Ludwig-Maximilians-University, Munich, 80539, Germany

[5]Department of Small Animal Medicine, University of Leipzig, Leipzig, 04103, Germany

[6]Department of Basic Medical Sciences, College of Veterinary Medicine, Purdue University, West Lafayette, IN, 47907, USA

[7]Small Animal Medicine and Surgery, University of Georgia, Athens, GA, 30602, USA

[8]Veterinary Sciences Centre, University College Dublin, Dublin, D04 V1W8, Ireland

[9]Department of Pathology, University of California, La Jolla, CA, 92093, USA

[10]Department of Population Health and Reproduction, University of California-Davis, Davis, CA, 95616, USA

[11]Pathobiology and Population Sciences, The Royal Veterinary College, North Mymms, Hertfordshire, AL9 7TA, UK

[12]Division of Clinical Neurology, University of Bern, Bern, 3001, Switzerland

[13]Division of Neurological Sciences, University of Bern, Bern, 3001, Switzerland

[+, ++]these author pairs contributed equally to this work

[*]**Corresponding author:** cord.droegemueller@vetsuisse.unibe.ch

Supplementary Video S1 LEMP-affected Leonberger showing typical gait abnormalities.

Supplementary Figure S1 MDS analysis for Leonbergers showing a scatter plot in which each point represents an individual. The axes correspond to a reduced representation of the data in two dimensions. Note that there is no obvious clustering neither of the 14 LEMP-affected dogs (shown in red) nor of the 186 controls (shown in blue).

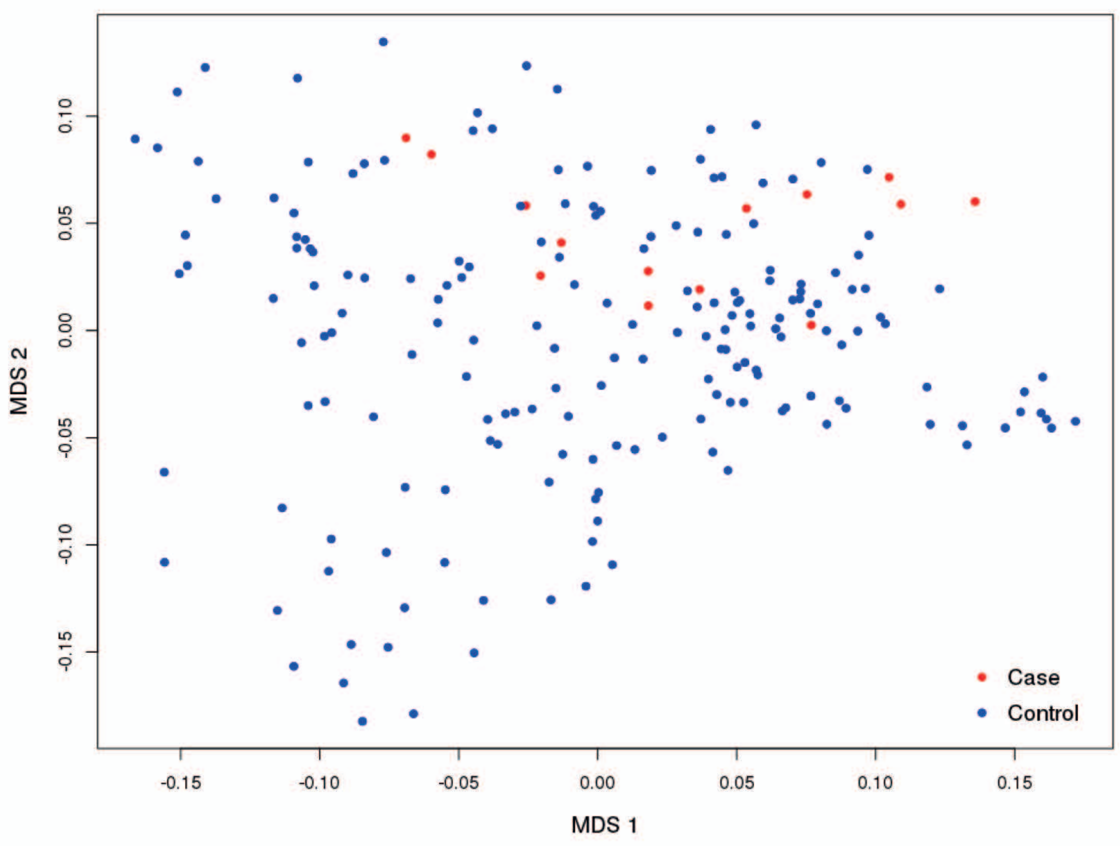

Supplementary Figure S2 Pedigree of 29 LEMP-diagnosed Leonbergers, including the two previously described cases[13], supports autosomal recessive inheritance.

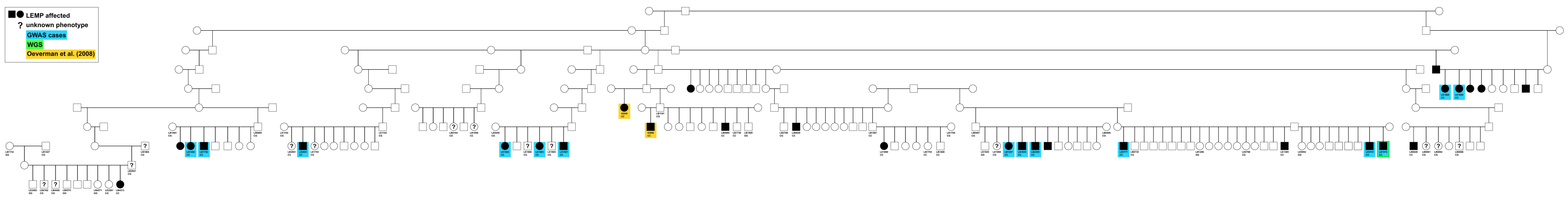

Supplementary Figure S3 (a) MDS analysis for Rottweilers showing a scatter plot in which each point represents an individual. The axes correspond to a reduced representation of the data in two dimensions. A genomic inflation factor (lambda) of 1.3 was observed. Note that there is no obvious clustering neither of the 3 LEMP-affected dogs (shown in red) nor of the 153 controls (shown in blue). (b) Manhattan plot for the GWAS indicates a signal with multiple associated SNPs on chromosome 18. The -log P-values for each SNP are plotted on the y-axis versus each canine chromosome on the x-axis. Inset: Corrected QQ-plot confirms that the actually observed P-values of the best associated markers have stronger association with the trait than expected by chance (null hypothesis, red line).

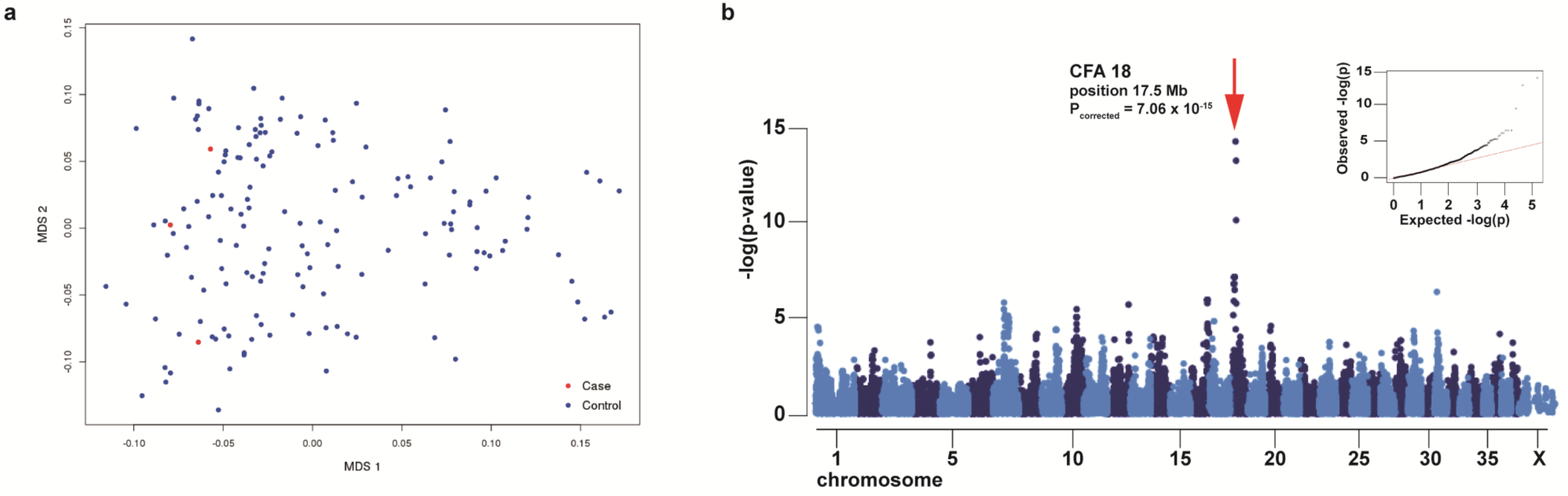

Supplementary Figure S4 Transverse section of the thoracic spinal cord (a) and peroneal nerve (b) in Leonberger case L8. (a) Hematoxylin & eosin staining. Scattered clear vacuoles are present in the white matter due to axonal degeneration with secondary dilation of myelin sheaths. Note that conspicuous white matter lesions as shown in Fig. 5 are lacking. (b) Masson-trichrome staining showing loss of myelinated axons in the peroneal nerve (asterisks).

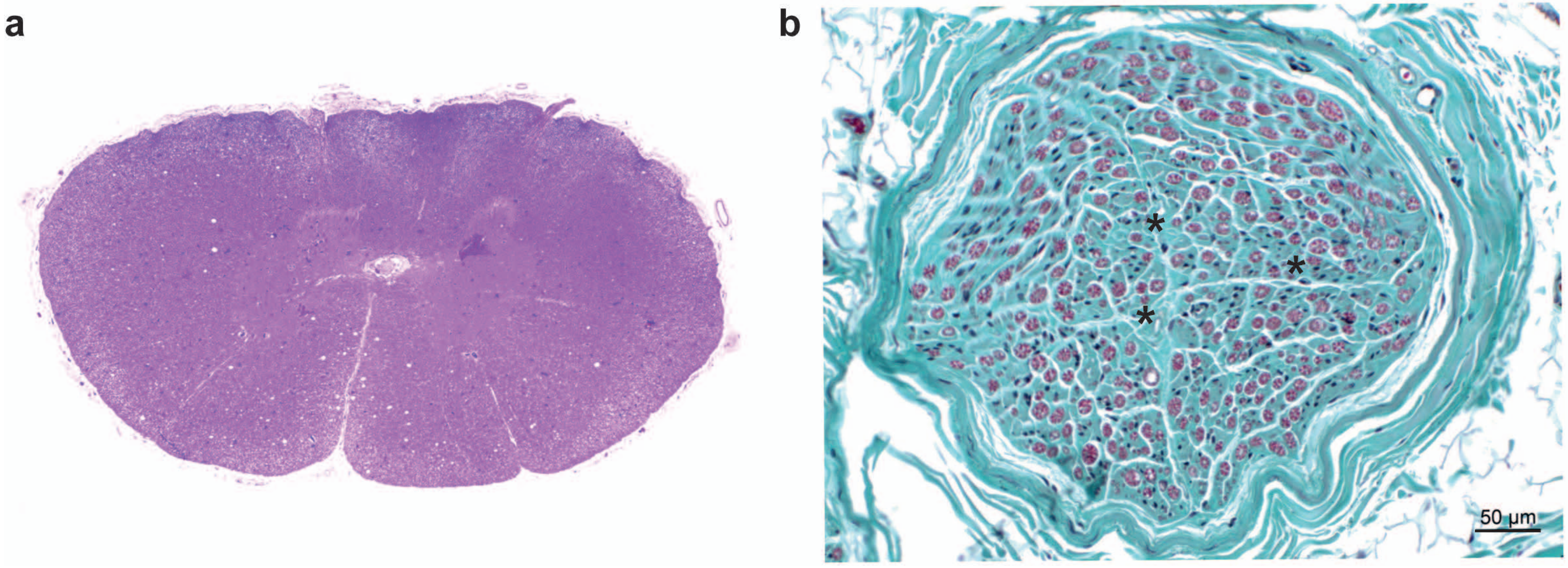

Supplementary Figure S5 Histopathology of the cerebral cortex (a) and spinal cord (b) of Leonberger case L7. Combined luxol fast blue/hematoxylin & eosin stain of paraffin sections. This case showed a well-defined plaque-like area of demyelination (indicated by arrows) in the corona radiata, but no lesion in the evaluated spinal cord section.

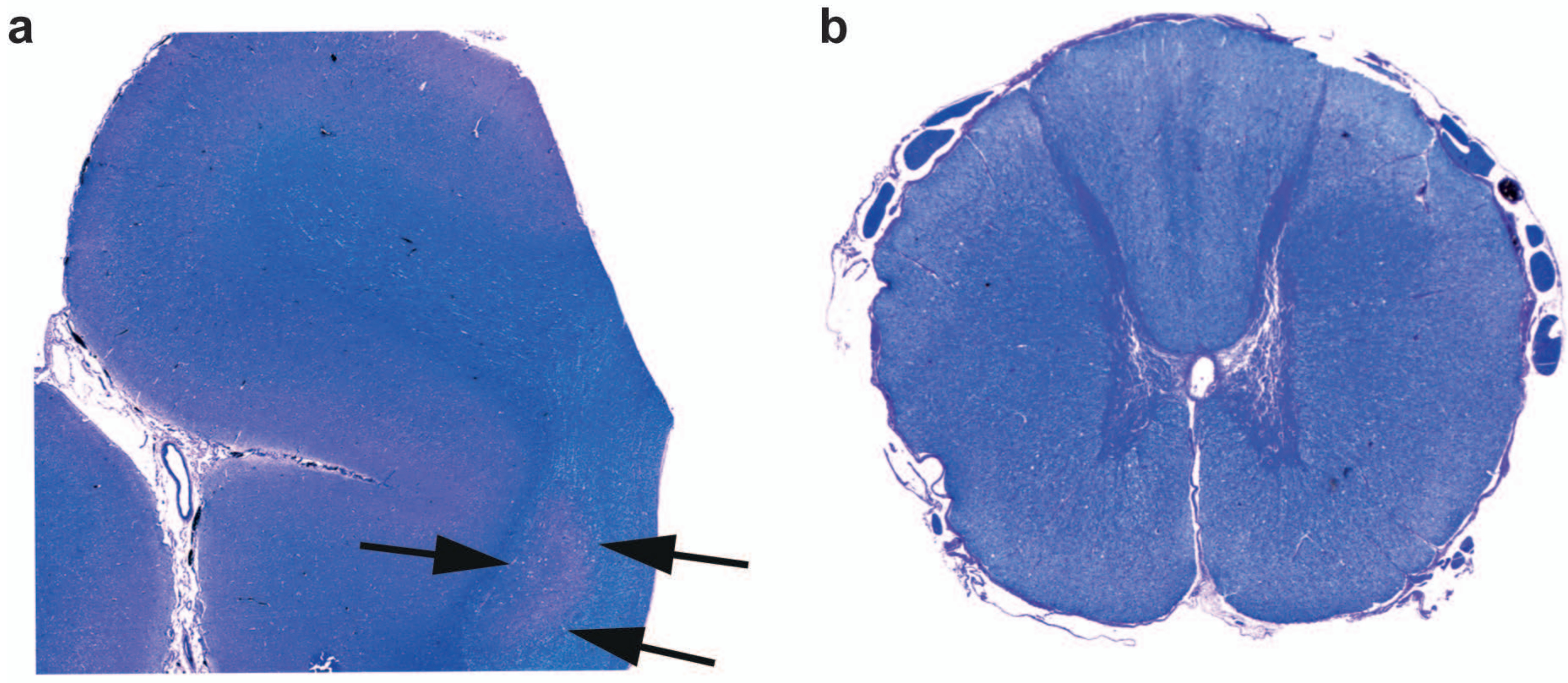

Supplementary Figure S6 Sequence of the canine NAPEPLD protein. The wild type protein sequence consists of 392 amino acids; the predicted mutant protein in Rottweilers with the frameshift variant consists of 301 amino acids and is shown below.

```
wt protein   1 MDENESNQSLMTSSQYPKEAVRKRQNSARSSVGSDSSRLSRKSFKLDYRL
            51 EEDVTKSKRGKDGRFVNPWPTWKDLSIPNVLRWLIMEKNHSGVPSSKEEL
           101 DKELPVLRPYFVDRPEAAGVSGAGLRVTWLGHATVLVEMDGLVLLTDPVF
           151 SPRASPWQRVGPRRFRRAPCSVAELPAVHAVVLSHNHYDHLDCGSVRALN
           201 ERFGPELRWFVPLGLLDWMQKCGCENVIELDWWEENCVPGHDSVTFVFTP
           251 AQHWCKRTLLDDNRVLWGSWAVLGPWSRFFFAGDTGYCSAFEEIGRRFGP
           301 FDLAAIPIGAYEPRWFMKYQHVDPEEAVRIHIDVQTKKSVAIHWGTFALA
           351 NEHYLEPPVKLSEALGRYGLNSEDFFVLKHGESRYLNADEEN

mt protein   1 MDENESNQSLMTSSQYPKEAVRKRQNSARSSVGSDSSRLSRKSFKLDYRL
            51 EEDVTKSKRGKDGRFVNPWPTWKDLSIPNVLRWLIMEKNHSGVPSSKEEL
           101 DKELPVLRPYFVDRPRGRGGERGGPAGHVAGPRHGPGGDGRAGAPHRPGV
           151 QPARLPVAARGAAALPAGALLGGRAARGAPRRPQPQPLRPPGLRLGARAQ
           201 RALRARAPLVCAPGPPGLDAEVRLRERDRAGLVGGELRARARQRHLRLYA
           251 RPALVQEDPPGRQQGSVGQLGRPGALEPLLFRRRHWLLLSFRRDRKEVWP
           301 F
```

Supplementary Figure S7 Structure of *NAPEPLD* gene. The genomic coordinates of the canine *NAPEPLD* gene on chromosome 18 are given based on the CanFam3.1 assembly. Please note the sequence gap downstream of exon 4. The unplaced contig sequence Un_JH373889 contains the missing exon 5 and partial sequence of intron 4.

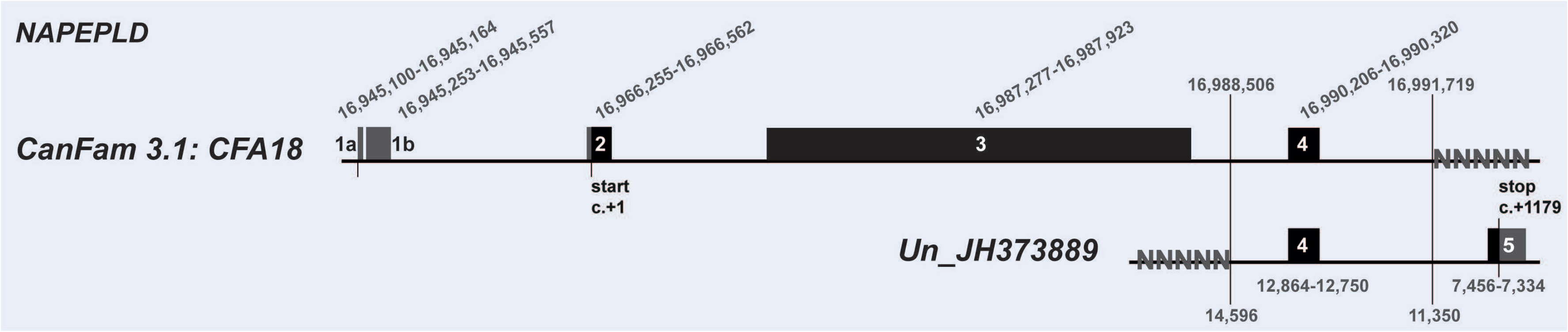

Table S1. Phenotypes and genotypes of 213 Leonbergers with detailed records.

Case no	LabID	Sex	Affection status (1: control; 2: case)	Age of onset of clinical signs (years)	Gait abnormalities	MRI	Necropsy	GWAS	Years of age at the time of genotyping (for controls only)	*NAPEPLD* : CFA18:g.16987520G>C	*ARHGEF10* : CFA16:g.54349199_54349208del	GJA9: CFA15:g.3863524_3863525delAG	References
L1	42840	f	2	1.5	yes	yes	(spinal cord, brainstem)			DD	NN	NN	Oevermann A. *et al* . (2008) *J Vet Intern Med* . **22(2)**, 467-71.
L2	42986	m	2	1.5	yes	yes	(spinal cord, brainstem)			DD	NN	NN	Oevermann A. *et al* . (2008) *J Vet Intern Med* . **22(2)**, 467-71.
L3	LB2385/LE1654/LE1462	m	2	2	yes	no	(spinal cord, midbrain, brainstem)	X		DD	DN	NN	
L4	LE2041	m	2	2.5	yes	yes	(spinal cord)	X		DD	NN	NN	
L5	LE1662	f	2	1.3	yes	no	(spinal cord, brainstem)	X		DD	NN	NN	
L6	LE1663/LE0455	f	2	1.4	yes	no	(spinal cord, brainstem)	X		DD	NN	NN	
L7	LE1680	f	2	1.3	yes	no	(spinal cord, cortex)			DD	NN	NN	
L8	LB4668	m	2	7.5	yes	no	(spinal cord, skeletal muscle, peripheral nerve)			DD	NN	NN	
L9	LB1257/LE1649	f	2	1.8	yes	no	not available	X		DD	DN	NN	
L10	LB1835/LE1653	m	2	2	yes	no	not available	X		DD	DN	NN	
L11	LE0777	m	2	4	yes	no	not available	X		DD	DN	NN	
L12	LB4313	f	2	1	yes	no	not available			DD	NN	NN	
L13	LB0989	m	2	2	yes	no	not available			DD	DN	NN	
L14	LB0029/LE0593	m	2	2	yes	no	not available			DD	DN	NN	
L15	LE0592/LB0026	m	2	1.5	yes	no	not available			DD	NN	NN	
L16	LE0741	m	2	3	yes	no	not available	X		DD	NN	NN	
L17	LE1326/LB1782	m	2	1.5	yes	no	not available	X		DD	NN	NN	
L18	LE1385	m	2	4	yes	no	not available			DD	NN	NN	
L19	LE1473/LB1919	f	2	3	yes	no	not available	X		DD	NN	NN	
L20	LE1659	f	2	2	yes	no	(spinal cord, cerebellum, brainstem)	X		DD	NN	NN	
L21	LE1660	f	2	2	yes	no	(spinal cord, cerebellum, brainstem)	X		DD	NN	NN	
L22	LE1661	m	2	1.3	yes	no	(spinal cord, cerebellum, brainstem)	X		DD	NN	NN	
L23	LE2068/LB1462	f	2	1.5	yes	no	not available	X		DD	NN	NN	
L24	LB0202/LE0696	f	2	2.5	yes	no	not available			DD	NN	NN	
L25	LB1712	f	2	2	yes	no	not available			DD	DN	NN	
L26	LB1755	f	2	3.5	yes	no	not available			DD	NN	NN	
L27	LB2536	m	2	4	yes	no	not available			DD	NN	NN	
	CFA007098	f	1					X	11.0	NN	NN	NN	
	LB0001	f	1					X	11.4	NN	NN	NN	
	LB0002	f	1					X	10.1	NN	NN	NN	
	LB0109	f	1					X	10.3	NN	NN	NN	
	LB0114	f	1					X	10.1	NN	NN	NN	
	LB0124	f	1					X	11.1	NN	NN	NN	
	LB0198	f	1					X	12.1	NN	NN	NN	
	LB0207	f	1					X	10.5	DN	NN	NN	
	LB0243	m	1					X	9.1	NN	NN	NN	
	LB0349	f	1					X	10.8	NN	NN	NN	
	LB0453	f	1					X	10.2	NN	NN	NN	
	LB0488	f	1					X	10.8	NN	NN	NN	

	LB0491	f	1					X	12.4	DN	NN	NN	
	LB0529	f	1					X	10.1	NN	NN	NN	
	LB0555	m	1					X	10.3	NN	NN	NN	
	LB0570	f	1					X	12.0	NN	NN	NN	
	LB0724	m	1					X	10.1	NN	NN	NN	
	LB0773	f	1					X	11.9	NN	NN	NN	
	LB0821	m	1					X	11.7	NN	NN	NN	
	LB0822	f	1					X	10.7	NN	NN	NN	
	LB0919	f	1					X	13.2	NN	NN	NN	
	LB1006	f	1					X	10.4	NN	NN	NN	
	LB1046	m	1					X	11.6	NN	NN	NN	
	LB1094	f	1					X	10.6	NN	NN	NN	
	LB1114	f	1					X	11.0	NN	NN	NN	
	LB1193	m	1					X	10.2	DN	NN	NN	
	LB1260	f	1					X	10.2	NN	NN	NN	
	LB1268	m	1					X	13.0	NN	NN	NN	
	LB1269	f	1					X	10.8	NN	NN	NN	
	LB1270	f	1					X	11.3	NN	NN	NN	
	LB1328	f	1					X	11.7	NN	NN	NN	
	LB1428	f	1					X	10.2	NN	NN	NN	
	LB1449	f	1					X	11.3	NN	NN	NN	
	LB1477	f	1					X	10.0	NN	NN	NN	
	LB1515	f	1					X	10.1	NN	NN	NN	
	LB1523	f	1					X	10.3	NN	NN	NN	
	LB1644	m	1					X	11.3	NN	NN	NN	
	LB1652	m	1					X	8.3	NN	NN	NN	
	LB1706	f	1					X	10.7	NN	NN	NN	
	LB2008	m	1					X	10.1	NN	NN	NN	
	LB2097	f	1					X	8.2	NN	NN	NN	
	LB2102	m	1					X	10.1	NN	NN	NN	
	LB2262	f	1					X	10.5	DN	NN	NN	
	LB2558	f	1					X	10.1	NN	NN	NN	
	LB2774	f	1					X	11.4	NN	NN	NN	
	LB2775	m	1					X	11.2	NN	NN	NN	
	LB2790	m	1					X	10.3	NN	NN	NN	
	LB3128	m	1					X	11.0	DN	NN	NN	
	LB3130	f	1					X	10.2	NN	NN	NN	
	LB3131	f	1					X	10.4	NN	NN	NN	
	LB3242	f	1					X	10.3	NN	NN	NN	
	LB3401	f	1					X	12.9	NN	NN	NN	
	LB4017	f	1					X	13.0	DN	NN	NN	

	LB4658	f	1					X	14.1	NN	NN	NN	
	LB4777	f	1					X	10.0	NN	NN	NN	
	LE0017	f	1					X	8.8	NN	NN	NN	
	LE0031	f	1					X	9.3	NN	NN	NN	
	LE0048	f	1					X	13.0	DN	NN	NN	
	LE0058	m	1					X	11.4	NN	NN	NN	
	LE0060	f	1					X	10.9	DN	NN	NN	
	LE0063	m	1					X	10.8	NN	NN	NN	
	LE0064	m	1					X	8.5	NN	NN	NN	
	LE0077	f	1					X	10.2	NN	NN	NN	
	LE0097	m	1					X	9.8	NN	NN	NN	
	LE0098	f	1					X	8.9	NN	NN	NN	
	LE0101	m	1					X	9.8	NN	NN	NN	
	LE0106	m	1					X	9.4	NN	NN	NN	
	LE0108	f	1					X	11.8	DN	NN	NN	
	LE0111	f	1					X	10.4	NN	NN	NN	
	LE0112	m	1					X	9.3	NN	NN	NN	
	LE0122	f	1					X	13.5	DN	NN	NN	
	LE0123	f	1					X	9.2	NN	NN	NN	
	LE0124	m	1					X	10.2	NN	NN	NN	
	LE0140	m	1					X	8.9	DN	NN	NN	
	LE0152	m	1					X	9.7	NN	NN	NN	
	LE0157	f	1					X	8.7	NN	NN	NN	
	LE0159	f	1					X	10.6	NN	NN	NN	
	LE0160	m	1					X	11.0	NN	NN	NN	
	LE0162	m	1					X	8.2	NN	NN	NN	
	LE0164	m	1					X	11.3	NN	NN	NN	
	LE0167	m	1					X	11.0	NN	NN	NN	
	LE0169	f	1					X	9.2	NN	NN	NN	
	LE0175	m	1					X	13.0	NN	NN	NN	
	LE0178	m	1					X	10.9	NN	NN	NN	
	LE0179	m	1					X	10.2	DN	NN	NN	
	LE0183	f	1					X	11.9	NN	NN	NN	
	LE0184	f	1					X	10.7	NN	NN	NN	
	LE0185	m	1					X	10.3	NN	NN	NN	
	LE0189	m	1					X	8.0	NN	NN	NN	
	LE0199	f	1					X	12.0	NN	NN	NN	
	LE0200	m	1					X	10.5	NN	NN	NN	
	LE0201	f	1					X	10.7	NN	NN	NN	
	LE0212	f	1					X	9.7	NN	NN	NN	
	LE0213	f	1					X	10.8	NN	NN	NN	

	LE0223	m	1					X	10.0	NN	NN	NN	
	LE0229	f	1					X	11.0	NN	NN	NN	
	LE0232	f	1					X	12.6	DN	NN	NN	
	LE0234	m	1					X	11.0	NN	NN	NN	
	LE0241	m	1					X	10.1	NN	NN	NN	
	LE0249	f	1					X	11.0	NN	NN	NN	
	LE0251	f	1					X	11.0	NN	NN	NN	
	LE0253	f	1					X	12.1	NN	NN	NN	
	LE0256	f	1					X	10.3	NN	NN	NN	
	LE0257	f	1					X	9.3	NN	NN	NN	
	LE0267	m	1					X	10.1	NN	NN	NN	
	LE0271	f	1					X	8.6	NN	NN	NN	
	LE0274	f	1					X	11.8	NN	NN	NN	
	LE0277	m	1					X	9.0	NN	NN	NN	
	LE0279	f	1					X	9.8	NN	NN	NN	
	LE0283	m	1					X	10.8	NN	NN	NN	
	LE0287	f	1					X	13.3	NN	NN	NN	
	LE0291	m	1					X	10.4	NN	NN	NN	
	LE0295	f	1					X	10.0	NN	NN	NN	
	LE0314	m	1					X	9.2	DN	NN	NN	
	LE0315	f	1					X	11.3	NN	NN	NN	
	LE0316	f	1					X	12.0	DN	NN	NN	
	LE0317	f	1					X	8.7	NN	NN	NN	
	LE0318	f	1					X	12.0	NN	NN	NN	
	LE0320	f	1					X	10.9	DN	NN	NN	
	LE0321	f	1					X	12.2	NN	NN	NN	
	LE0337	m	1					X	9.3	DN	NN	NN	
	LE0339	m	1					X	12.0	NN	NN	NN	
	LE0340	f	1					X	8.9	NN	NN	NN	
	LE0349	m	1					X	8.3	DN	NN	NN	
	LE0368	f	1					X	10.7	NN	NN	NN	
	LE0372	m	1					X	8.3	NN	NN	NN	
	LE0376	m	1					X	9.0	DN	NN	NN	
	LE0377	m	1					X	8.9	NN	NN	NN	
	LE0406	f	1					X	9.0	NN	NN	NN	
	LE0409	m	1					X	11.0	NN	NN	NN	
	LE0413	f	1					X	11.0	NN	NN	NN	
	LE0436	m	1					X	10.5	DN	NN	NN	
	LE0439	f	1					X	11.1	NN	NN	NN	
	LE0459	m	1					X	10.2	NN	NN	NN	
	LE0477	m	1					X	12.3	DN	NN	NN	

	LE0484	m	1					X	9.5	NN	NN	NN	
	LE0487	f	1					X	10.8	NN	NN	NN	
	LE0489	f	1					X	12.6	NN	NN	NN	
	LE0494	m	1					X	8.2	NN	NN	NN	
	LE0499	f	1					X	12.6	DN	NN	NN	
	LE0504	m	1					X	9.1	NN	NN	NN	
	LE0505	m	1					X	10.7	NN	NN	NN	
	LE0517	m	1					X	9.8	NN	NN	NN	
	LE0522	m	1					X	8.5	NN	NN	NN	
	LE0535	f	1					X	10.6	NN	NN	NN	
	LE0539	f	1					X	10.9	NN	NN	NN	
	LE0541	f	1					X	9.0	NN	NN	NN	
	LE0546	f	1					X	10.1	NN	NN	NN	
	LE0549	m	1					X	8.5	DN	NN	NN	
	LE0552	m	1					X	11.0	NN	NN	NN	
	LE0560	f	1					X	8.7	NN	NN	NN	
	LE0581/LB0125	f	1					X	12.8	NN	NN	NN	
	LE0595/LB0031	m	1					X	9.9	NN	NN	NN	
	LE0617	f	1					X	8.2	DN	NN	NN	
	LE0620	f	1					X	11.1	NN	NN	NN	
	LE0667	f	1					X	9.0	NN	NN	NN	
	LE0714	f	1					X	12.0	NN	NN	NN	
	LE0766	m	1					X	9.9	NN	NN	NN	
	LE0787	m	1					X	10.1	NN	NN	NN	
	LE0815	m	1					X	11.7	NN	NN	NN	
	LE0818	m	1					X	10.7	NN	NN	NN	
	LE0838	m	1					X	10.7	NN	NN	NN	
	LE0868	m	1					X	10.2	NN	NN	NN	
	LE0871	m	1					X	10.0	DN	NN	NN	
	LE0882	f	1					X	12.8	NN	NN	NN	
	LE0891	m	1					X	9.9	NN	NN	NN	
	LE0894	f	1					X	8.2	NN	NN	NN	
	LE0910	m	1					X	9.9	NN	NN	NN	
	LE0913	m	1					X	9.8	NN	NN	NN	
	LE0948	f	1					X	9.5	NN	NN	NN	
	LE0978	m	1					X	12.6	DN	NN	NN	
	LE1035	m	1					X	11.7	NN	NN	NN	
	LE1139	m	1					X	10.1	NN	NN	NN	
	LE1144	m	1					X	10.7	NN	NN	NN	
	LE1178	m	1					X	9.0	NN	NN	NN	
	LE1188	f	1					X	10.7	NN	NN	NN	

	LE1200	m	1					X	8.8	NN	NN	NN	
	LE1329	m	1					X	12.3	NN	NN	NN	
	LE1476	m	1					X	8.4	NN	NN	NN	
	LE1685	m	1					X	10.0	DN	NN	NN	
	LE1686	m	1					X	9.4	NN	NN	NN	
	LE1980	m	1					X	12.6	DN	NN	NN	
	LE1981	m	1					X	11.4	NN	NN	NN	
	LEO_BI_181360	f	1					X	10.5	NN	NN	NN	
	LEO_BI_279651_STARKEY _LB-NC-18	f	1					X	10.4	NN	NN	NN	
	LEO_BI_279658_STARKEY _LB-OS-07	f	1					X	8.8	NN	NN	NN	

Table S2. Sample designations and breed information on 206 individuals with genome sequences.

Sample ID	Breed	Study Accession	Sample Accession	Remarks
AC023	Australian Cattle dog	PRJEB16012	SAMEA104091560	
AC065	Australian Cattle dog	PRJEB13468	SAMEA3928139	
AC108	Australian Cattle dog	PRJEB16012	SAMEA4504820	
AD009	Alpine Dachsbracke	PRJEB14840	SAMEA4346711	
AM007	Alaskan Malamute	PRJEB16012	SAMEA104091571	
AR001	Australian Terrier	PRJEB16012	SAMEA4504840	
AS006	American Staffordshire Terrier	PRJEB16012	SAMEA4504831	
BB011	Berger Blanc Suisse	PRJEB16012	SAMEA4506887	
BC0480	Border Collie	PRJEB16012	SAMEA104091558	
BC272	Border Collie	PRJEB4544	SAMEA2177732	
BC273	Border Collie	PRJEB16012	SAMEA4505490	
BC485	Border Collie	PRJEB16012	SAMEA104125112	
BC518	Border Collie	PRJEB16012	SAMEA104125113	
BC555	Border Collie	PRJEB16012	SAMEA104125114	
BC597	Border Collie	PRJEB16012	SAMEA104125115	
BD016	Bearded Collie	PRJEB13468	SAMEA3928140	
BD036	Bearded Collie	PRJEB16012	SAMEA4505492	
BD052	Bearded Collie	PRJEB16012	SAMEA4504824	
BD085	Bearded Collie	PRJEB16012	SAMEA4505493	
BD089	Bearded Collie	PRJEB16012	SAMEA4504827	
BD091	Bearded Collie	PRJEB16012	SAMEA4505496	
BD093	Bearded Collie	PRJEB16012	SAMEA4504832	
BD098	Bearded Collie	PRJEB16012	SAMEA4504839	
BE020	Beagle	PRJEB5500	SAMEA2376414	
BG064	Bavarian Hound	PRJEB16012	SAMEA104091562	
BH003	Basset	PRJEB16012	SAMEA4509488	
BT007	Miniature Bullterrier	PRJEB16012	SAMEA4506897	
BT012	Bullterrier	PRJEB16012	SAMEA104125116	
BU002	Bullmastiff	PRJEB16012	SAMEA103949042	
CE073	Cairn Terrier	PRJEB16012	SAMEA104091555	
CH019	Chihuahua	PRJEB13139	SAMEA3905753	
CK006	Cavalier King Charles Spaniel	PRJEB16012	SAMEA104091570	
CK023	Cavalier King Charles Spaniel	PRJEB16012	SAMEA104091569	
CP003	Cocker Spaniel	PRJEB16012	SAMEA4506900	
CR039	Curly Coated Retriever	PRJEB16012	SAMEA104091556	
CW011	Chow Chow	PRJEB16012	SAMEA104091566	
DD116	Great Dane	PRJEB16012	SAMEA104091557	
DH0117	Dachshund	PRJEB16012	SAMEA104091567	
DH098	Dachshund	PRJEB7736	SAMEA3121338	
DH126	Dachshund	PRJEB16012	SAMEA104125117	
DO159	Doberman Pinscher	PRJEB16012	SAMEA4509491	
DO242	Doberman Pinscher	PRJEB16012	SAMEA4505489	
DO263	Doberman Pinscher	PRJEB16012	SAMEA4509492	
DQ1	Chinese indigenous dogs	PRJNA266585	Bai B. *et al* . (2015) Nucleic Acids Res. **43,** D777-83.	
DQ10	Chinese indigenous dogs	PRJNA266585	Bai B. *et al* . (2015) Nucleic Acids Res. **43,** D777-83.	
DQ2	Chinese indigenous dogs	PRJNA266585	Bai B. *et al* . (2015) Nucleic Acids Res. **43,** D777-83.	
DQ3	Chinese indigenous dogs	PRJNA266585	Bai B. *et al* . (2015) Nucleic Acids Res. **43,** D777-83.	
DQ4	Chinese indigenous dogs	PRJNA266585	Bai B. *et al* . (2015) Nucleic Acids Res. **43,** D777-83.	
DQ5	Chinese indigenous dogs	PRJNA266585	Bai B. *et al* . (2015) Nucleic Acids Res. **43,** D777-83.	
DQ6	Chinese indigenous dogs	PRJNA266585	Bai B. *et al* . (2015) Nucleic Acids Res. **43,** D777-83.	
DQ7	Chinese indigenous dogs	PRJNA266585	Bai B. *et al* . (2015) Nucleic Acids Res. **43,** D777-83.	
DQ8	Chinese indigenous dogs	PRJNA266585	Bai B. *et al* . (2015) Nucleic Acids Res. **43,** D777-83.	
DQ9	Chinese indigenous dogs	PRJNA266585	Bai B. *et al* . (2015) Nucleic Acids Res. **43,** D777-83.	
DS032	German Shepherd/Mixed breed	PRJEB14110	SAMEA3994036	
DS041	German Shepherd/Mixed breed	PRJEB14110	SAMEA3994037	
DS042	German Shepherd/Mixed breed	PRJEB14110	SAMEA3994038	
DS043	German Shepherd	PRJEB16012	SAMEA4506895	
DS051	German Shepherd	PRJEB16012	SAMEA72802168	
DS053	German Shepherd	PRJEB16012	SAMEA72802918	
EL565	Elo	PRJEB16012	SAMEA4506890	
EN091	Entlebucher Sennenhund	PRJEB16012	SAMEA4504828	
EN154	Entlebucher Sennenhund	PRJEB16012	SAMEA4505497	
EN221	Entlebucher Sennenhund	PRJEB16012	SAMEA4504829	
EN247	Entlebucher Sennenhund	PRJEB16012	SAMEA4505498	
EN261	Entlebucher Sennenhund	PRJEB16012	SAMEA4504830	
EN262	Entlebucher Sennenhund	PRJEB16012	SAMEA4505499	
EN263	Entlebucher Sennenhund	PRJEB16012	SAMEA4505500	
EN264	Entlebucher Sennenhund	PRJEB16012	SAMEA4505501	
EU008	Eurasier	PRJEB6079	SAMEA2446720	
EU035	Eurasier	PRJEB16012	SAMEA4506889	
FB046	French Bulldog	PRJEB13468	SAMEA3928146	
FB065	French Bulldog	PRJEB16012	SAMEA4504835	
GR0855	Golden Retriever	PRJEB16012	SAMEA4504838	
GR0859	Golden Retriever	PRJEB16012	SAMEA4506899	
GR0860	Golden Retriever	PRJEB16012	SAMEA4509487	
GR0892	Golden Retriever	PRJEB16012	SAMEA104016307	
GR1078	Golden Retriever	PRJEB16012	SAMEA104091572	
GS1	German Shepherd	PRJNA266585	Bai B. *et al* . (2015) Nucleic Acids Res. **43,** D777-83.	
GS10	German Shepherd	PRJNA266585	Bai B. *et al* . (2015) Nucleic Acids Res. **43,** D777-83.	
GS104	German Spitz	PRJEB16012	SAMEA104105252	
GS2	German Shepherd	PRJNA266585	Bai B. *et al* . (2015) Nucleic Acids Res. **43,** D777-83.	

GS3	German Shepherd	PRJNA266585	Bai B. *et al* . (2015) Nucleic Acids Res. **43,** D777-83.	
GS4	German Shepherd	PRJNA266585	Bai B. *et al* . (2015) Nucleic Acids Res. **43,** D777-83.	
GS5	German Shepherd	PRJNA266585	Bai B. *et al* . (2015) Nucleic Acids Res. **43,** D777-83.	
GS6	German Shepherd	PRJNA266585	Bai B. *et al* . (2015) Nucleic Acids Res. **43,** D777-83.	
GS7	German Shepherd	PRJNA266585	Bai B. *et al* . (2015) Nucleic Acids Res. **43,** D777-83.	
GS8	German Shepherd	PRJNA266585	Bai B. *et al* . (2015) Nucleic Acids Res. **43,** D777-83.	
GS9	German Shepherd	PRJNA266585	Bai B. *et al* . (2015) Nucleic Acids Res. **43,** D777-83.	
GW004	German Wirehaired	PRJEB13468	SAMEA3928144	
GY432	Greyhound	PRJEB16012	SAMEA104125118	
HT001	Heideterrier	PRJEB16012	SAMEA103135918	
HW1706	Hovawart	PRJEB16012	SAMEA4506894	
IT221	Irish Terrier	PRJEB13468	SAMEA3928141	
JR0034	Jack Russell Terrier	PRJEB16012	SAMEA104125119	
JT007	German Hunting Terrier	PRJEB16012	SAMEA104125120	
KF042	Kromfohrländer	PRJEB6076	SAMEA2446055	
KKH2684	Siberian Husky	PRJEB10823	SAMEA3539249	
KM1	Kunming Dog	PRJNA266585	Bai B. *et al* . (2015) Nucleic Acids Res. **43,** D777-83.	
KM10	Kunming Dog	PRJNA266585	Bai B. *et al* . (2015) Nucleic Acids Res. **43,** D777-83.	
KM2	Kunming Dog	PRJNA266585	Bai B. *et al* . (2015) Nucleic Acids Res. **43,** D777-83.	
KM3	Kunming Dog	PRJNA266585	Bai B. *et al* . (2015) Nucleic Acids Res. **43,** D777-83.	
KM4	Kunming Dog	PRJNA266585	Bai B. *et al* . (2015) Nucleic Acids Res. **43,** D777-83.	
KM5	Kunming Dog	PRJNA266585	Bai B. *et al* . (2015) Nucleic Acids Res. **43,** D777-83.	
KM6	Kunming Dog	PRJNA266585	Bai B. *et al* . (2015) Nucleic Acids Res. **43,** D777-83.	
KM7	Kunming Dog	PRJNA266585	Bai B. *et al* . (2015) Nucleic Acids Res. **43,** D777-83.	
KM8	Kunming Dog	PRJNA266585	Bai B. *et al* . (2015) Nucleic Acids Res. **43,** D777-83.	
KM9	Kunming Dog	PRJNA266585	Bai B. *et al* . (2015) Nucleic Acids Res. **43,** D777-83.	
LA1869	Labrador Retriever	PRJEB16012	SAMEA4504834	
LA2382	Labrador Retriever	PRJEB16012	SAMEA104125075	
LA2443	Labrador Retriever	PRJEB16012	SAMEA104091568	
LA882	Labrador Retriever	PRJEB5874	SAMEA2417015	
LA900	Labrador Retriever	PRJEB5875	SAMEA2417016	
LB111	Leonberger	PRJEB16012	SAMEA47266168	
**LB1919**	**Leonberger**	**PRJEB16012**	**SAMEA103935360**	**Affected dog carrying a *NAPEPLD* variant**
LJ1	Chinese indigenous dogs	PRJNA266585	Bai B. *et al* . (2015) Nucleic Acids Res. **43,** D777-83.	
LJ10	Chinese indigenous dogs	PRJNA266585	Bai B. *et al* . (2015) Nucleic Acids Res. **43,** D777-83.	
LJ2	Chinese indigenous dogs	PRJNA266585	Bai B. *et al* . (2015) Nucleic Acids Res. **43,** D777-83.	
LJ3	Chinese indigenous dogs	PRJNA266585	Bai B. *et al* . (2015) Nucleic Acids Res. **43,** D777-83.	
LJ4	Chinese indigenous dogs	PRJNA266585	Bai B. *et al* . (2015) Nucleic Acids Res. **43,** D777-83.	
LJ5	Chinese indigenous dogs	PRJNA266585	Bai B. *et al* . (2015) Nucleic Acids Res. **43,** D777-83.	
LJ6	Chinese indigenous dogs	PRJNA266585	Bai B. *et al* . (2015) Nucleic Acids Res. **43,** D777-83.	
LJ7	Chinese indigenous dogs	PRJNA266585	Bai B. *et al* . (2015) Nucleic Acids Res. **43,** D777-83.	
LJ8	Chinese indigenous dogs	PRJNA266585	Bai B. *et al* . (2015) Nucleic Acids Res. **43,** D777-83.	
LJ9	Chinese indigenous dogs	PRJNA266585	Bai B. *et al* . (2015) Nucleic Acids Res. **43,** D777-83.	
LN048	Landseer	PRJEB9437	SAMEA3402917	
LN47	Border Collie	PRJEB16012	SAMEA4504821	
LR1030	Lagotto Romagnolo	PRJEB16012	SAMEA4509490	
LR390	Lagotto Romagnolo	PRJEB13468	SAMEA3928142	
LR433	Lagotto Romagnolo	PRJEB16012	SAMEA4505491	
LR494	Lagotto Romagnolo	PRJEB16012	SAMEA4505494	
LR753	Lagotto Romagnolo	PRJEB16012	SAMEA4504833	
MA008	Malinois	PRJEB16012	SAMEA4504823	
MA0163	Malinois	PRJEB16012	SAMEA104032048	
MA094	Malinois	PRJEB16012	SAMEA4504826	
MA142	Malinois	PRJEB16012	SAMEA104125121	
MA152	Malinois	PRJEB16012	SAMEA4506898	
MA324	Malinois	PRJEB16012	SAMEA104125122	
MI016	Mixed Breed	PRJEB16012	SAMEA4506893	
NW062	Norwich Terrier	PRJEB16012	SAMEA104091550	
NW152	Norwich Terrier	PRJEB16012	SAMEA104091551	
NW206	Norwich Terrier	PRJEB16012	SAMEA104091552	
NW255	Norwich Terrier	PRJEB16012	SAMEA104091553	
OS001	Old English Sheepdog	PRJEB16012	SAMEA4504841	
PE002	Perro de Agua Español	PRJEB7903	SAMEA3164479	
PL116	Poodle	PRJEB16012	SAMEA4506891	
**RO002**	**Rottweiler**	**PRJEB7735**	**SAMEA3121337**	**Affected dog carrying a *NAPEPLD* variant**
RR098	Rhodesian Ridgeback	PRJEB16012	SAMEA104091554	
RR123	Rhodesian Ridgeback	PRJEB16012	SAMEA4504822	
RR224	Rhodesian Ridgeback	PRJEB16012	SAMEA4505495	
SG005	Sloughi	PRJEB13468	SAMEA3928143	
SG006	Sloughi	PRJEB16012	SAMEA4506885	
SG008	Sloughi	PRJEB16012	SAMEA4506888	
SH008	Greater Swiss Mountain Dog	PRJEB16012	SAMEA4504837	
SL006	Saluki	PRJEB16012	SAMEA4504825	
SS004	Shetland Sheepdog	PRJEB16012	SAMEA104091573	
SY001	Alaskan Husky	PRJEB9590	SAMEA3449656	
SY018	Alaskan Husky	PRJEB9591	SAMEA3449657	
SY046	Siberian Husky	PRJEB16012	SAMEA104091559	
TA001	Airedale Terrier	PRJEB16012	SAMEA4506896	
TM1	Tibetan Mastiff	PRJNA266585	Bai B. *et al* . (2015) Nucleic Acids Res. **43,** D777-83.	
TM10	Tibetan Mastiff	PRJNA266585	Bai B. *et al* . (2015) Nucleic Acids Res. **43,** D777-83.	
TM2	Tibetan Mastiff	PRJNA266585	Bai B. *et al* . (2015) Nucleic Acids Res. **43,** D777-83.	
TM3	Tibetan Mastiff	PRJNA266585	Bai B. *et al* . (2015) Nucleic Acids Res. **43,** D777-83.	
TM4	Tibetan Mastiff	PRJNA266585	Bai B. *et al* . (2015) Nucleic Acids Res. **43,** D777-83.	

TM5	Tibetan Mastiff	PRJNA266585	Bai B. *et al* . (2015) Nucleic Acids Res. **43,** D777-83.	
TM6	Tibetan Mastiff	PRJNA266585	Bai B. *et al* . (2015) Nucleic Acids Res. **43,** D777-83.	
TM7	Tibetan Mastiff	PRJNA266585	Bai B. *et al* . (2015) Nucleic Acids Res. **43,** D777-83.	
TM8	Tibetan Mastiff	PRJNA266585	Bai B. *et al* . (2015) Nucleic Acids Res. **43,** D777-83.	
TM9	Tibetan Mastiff	PRJNA266585	Bai B. *et al* . (2015) Nucleic Acids Res. **43,** D777-83.	
WE006	Weimaraner	PRJEB16012	SAMEA4506902	
WH083	Whippet	PRJEB13468	SAMEA4506886	
WW174	West Highland White Terrier	PRJEB16012	SAMEA4506901	
WW361	West Highland White Terrier	PRJEB16012	SAMEA4504842	
WW362	West Highland White Terrier	PRJEB16012	SAMEA4509489	
WW363	West Highland White Terrier	PRJEB16012	SAMEA4504843	
WW364	West Highland White Terrier	PRJEB16012	SAMEA4504836	
WW558	West Highland White Terrier	PRJEB16012	SAMEA104091561	
WW64	West Highland White Terrier	PRJEB13723	SAMEA3940300	
YJ1	Chinese indigenous dogs	PRJNA266585	Bai B. *et al* . (2015) Nucleic Acids Res. **43,** D777-83.	
YJ2	Chinese indigenous dogs	PRJNA266585	Bai B. *et al* . (2015) Nucleic Acids Res. **43,** D777-83.	
YJ3	Chinese indigenous dogs	PRJNA266585	Bai B. *et al* . (2015) Nucleic Acids Res. **43,** D777-83.	
YJ4	Chinese indigenous dogs	PRJNA266585	Bai B. *et al* . (2015) Nucleic Acids Res. **43,** D777-83.	
YJ5	Chinese indigenous dogs	PRJNA266585	Bai B. *et al* . (2015) Nucleic Acids Res. **43,** D777-83.	
YJ6	Chinese indigenous dogs	PRJNA266585	Bai B. *et al* . (2015) Nucleic Acids Res. **43,** D777-83.	
YJ7	Chinese indigenous dogs	PRJNA266585	Bai B. *et al* . (2015) Nucleic Acids Res. **43,** D777-83.	
YJ9	Chinese indigenous dogs	PRJNA266585	Bai B. *et al* . (2015) Nucleic Acids Res. **43,** D777-83.	
YT003	Yorkshire Terrier	PRJEB13468	SAMEA3928145	
ZS14	Landseer	PRJEB7734	SAMEA3121328	
ZS14_2	Pomeranian	PRJEB16012	SAMEA4506892	
WO001_895	Wolf	PRJEB16012	SAMEA104091563	
WO002_732	Wolf	PRJEB16012	SAMEA104091564	
WO003_636	Wolf	PRJEB16012	SAMEA104091565	
BJ001	Basenji	PRJEB16012	SAMEA104283473	
BJ002	Basenji	PRJEB16012	SAMEA104283472	
BJ003	Basenji	PRJEB16012	SAMEA104283471	
BJ004	Basenji	PRJEB16012	SAMEA104283470	
BJ005	Basenji	PRJEB16012	SAMEA104283469	
BJ006	Basenji	PRJEB16012	SAMEA104283468	
IT0390	Irish Terrier	PRJEB16012	SAMEA104283467	
WE008	Weimaraner	PRJEB16012	SAMEA104283466	
DS064	German Shepherd	PRJEB16012	SAMEA104283465	
AC046	Australian Cattle Dog	PRJEB16012	SAMEA104283464	
JT011	Jagdterrier	PRJEB16012	SAMEA104283460	
AU178	Australian Shepherd	PRJEB16012	SAMEA104283461	
LR538	Lagotto Romagnolo	PRJEB16012	SAMEA104283462	
LR654	Lagotto Romagnolo	PRJEB16012	SAMEA104283463	

Table S3. List of 32 private sequence variants of the sequenced LEMP-affected Leonberger in the critical region on CFA18.

#CHROM	POS	REF	ALT	EFFECT	IMPACT	GENE	FEATURE	BIOTYPE	HGVS_C
18	16784506	T	C	intron_variant	MODIFIER	TRNAF-GAA	transcript	pseudogene	n.146+4563047T>C
18	16784506	T	C	intron_variant	MODIFIER	TRNAV-UAC	transcript	pseudogene	n.220-2032616A>G
18	16784506	T	C	intron_variant	MODIFIER	SLC26A5	transcript	protein_coding	c.-286+602T>C
18	16945736	A	T	intron_variant	MODIFIER	TRNAF-GAA	transcript	pseudogene	n.146+4724277A>T
18	16945736	A	T	intron_variant	MODIFIER	TRNAV-UAC	transcript	pseudogene	n.220-2193846T>A
18	16945736	A	T	intron_variant	MODIFIER	NAPEPLD	transcript	protein_coding	c.-14+179A>T
**18**	**16987520**	**G**	**C**	**missense_variant**	**MODERATE**	**NAPEPLD**	**transcript**	**protein_coding**	**c.538G>C**
18	16987520	G	C	intron_variant	MODIFIER	TRNAF-GAA	transcript	pseudogene	n.146+4766061G>C
18	16987520	G	C	intron_variant	MODIFIER	TRNAV-UAC	transcript	pseudogene	n.220-2235630C>G
18	17150356	G	T	intron_variant	MODIFIER	TRNAF-GAA	transcript	pseudogene	n.146+4928897G>T
18	17150356	G	T	intron_variant	MODIFIER	TRNAV-UAC	transcript	pseudogene	n.220-2398466C>A
18	17150356	G	T	intron_variant	MODIFIER	FBXL13	transcript	protein_coding	c.1279-5380G>T
18	17150356	G	T	intron_variant	MODIFIER	FBXL13	transcript	protein_coding	c.1144-5380G>T
18	17150356	G	T	intron_variant	MODIFIER	FBXL13	transcript	protein_coding	c.1270-5380G>T
18	17181890	TTA	T	intron_variant	MODIFIER	TRNAF-GAA	transcript	pseudogene	n.146+4960432_146+4960433delTA
18	17181890	TTA	T	intron_variant	MODIFIER	TRNAV-UAC	transcript	pseudogene	n.220-2430002_220-2430001delTA
18	17181890	TTA	T	intron_variant	MODIFIER	FBXL13	transcript	protein_coding	c.1906-7148_1906-7147delTA
18	17181890	TTA	T	intron_variant	MODIFIER	FBXL13	transcript	protein_coding	c.1771-7148_1771-7147delTA
18	17181890	TTA	T	intron_variant	MODIFIER	FBXL13	transcript	protein_coding	c.1897-7148_1897-7147delTA
18	17181890	TTA	T	intron_variant	MODIFIER	FBXL13	transcript	protein_coding	c.1778+18608_1778+18609delTA
18	17187149	T	A	intron_variant	MODIFIER	TRNAF-GAA	transcript	pseudogene	n.146+4965690T>A
18	17187149	T	A	intron_variant	MODIFIER	TRNAV-UAC	transcript	pseudogene	n.220-2435259A>T
18	17187149	T	A	intron_variant	MODIFIER	FBXL13	transcript	protein_coding	c.1906-1890T>A
18	17187149	T	A	intron_variant	MODIFIER	FBXL13	transcript	protein_coding	c.1771-1890T>A
18	17187149	T	A	intron_variant	MODIFIER	FBXL13	transcript	protein_coding	c.1897-1890T>A
18	17187149	T	A	intron_variant	MODIFIER	FBXL13	transcript	protein_coding	c.1778+23866T>A
18	17218251	T	A	intron_variant	MODIFIER	TRNAF-GAA	transcript	pseudogene	n.146+4996792T>A
18	17218251	T	A	intron_variant	MODIFIER	TRNAV-UAC	transcript	pseudogene	n.220-2466361A>T
18	17218251	T	A	intron_variant	MODIFIER	FBXL13	transcript	protein_coding	c.2375+3443T>A
18	17218251	T	A	intron_variant	MODIFIER	FBXL13	transcript	protein_coding	c.2240+3443T>A
18	17218251	T	A	intron_variant	MODIFIER	FBXL13	transcript	protein_coding	c.2366+3443T>A
18	17218251	T	A	intron_variant	MODIFIER	FBXL13	transcript	protein_coding	c.2288+3443T>A
18	17218251	T	A	intron_variant	MODIFIER	FBXL13	transcript	protein_coding	c.2153+3443T>A
18	17218251	T	A	intron_variant	MODIFIER	FBXL13	transcript	protein_coding	c.1942+3443T>A
18	17219701	C	G	intron_variant	MODIFIER	TRNAF-GAA	transcript	pseudogene	n.146+4998242C>G
18	17219701	C	G	intron_variant	MODIFIER	TRNAV-UAC	transcript	pseudogene	n.220-2467811G>C
18	17219701	C	G	intron_variant	MODIFIER	FBXL13	transcript	protein_coding	c.2375+4893C>G
18	17219701	C	G	intron_variant	MODIFIER	FBXL13	transcript	protein_coding	c.2240+4893C>G
18	17219701	C	G	intron_variant	MODIFIER	FBXL13	transcript	protein_coding	c.2366+4893C>G
18	17219701	C	G	intron_variant	MODIFIER	FBXL13	transcript	protein_coding	c.2288+4893C>G
18	17219701	C	G	intron_variant	MODIFIER	FBXL13	transcript	protein_coding	c.2153+4893C>G
18	17219701	C	G	intron_variant	MODIFIER	FBXL13	transcript	protein_coding	c.1942+4893C>G
18	17230470	A	T	intron_variant	MODIFIER	TRNAF-GAA	transcript	pseudogene	n.146+5009011A>T
18	17230470	A	T	intron_variant	MODIFIER	TRNAV-UAC	transcript	pseudogene	n.220-2478580T>A
18	17230470	A	T	intron_variant	MODIFIER	FAM185A	transcript	protein_coding	c.1082-1085T>A
18	17232001	T	A	intron_variant	MODIFIER	TRNAF-GAA	transcript	pseudogene	n.146+5010542T>A
18	17232001	T	A	intron_variant	MODIFIER	TRNAV-UAC	transcript	pseudogene	n.220-2480111A>T
18	17232001	T	A	intron_variant	MODIFIER	FAM185A	transcript	protein_coding	c.1082-2616A>T
18	17244679	T	C	intron_variant	MODIFIER	TRNAF-GAA	transcript	pseudogene	n.146+5023220T>C
18	17244679	T	C	intron_variant	MODIFIER	TRNAV-UAC	transcript	pseudogene	n.220-2492789A>G
18	17244679	T	C	intron_variant	MODIFIER	FAM185A	transcript	protein_coding	c.946+294A>G
18	17275577	A	T	intron_variant	MODIFIER	TRNAF-GAA	transcript	pseudogene	n.146+5054118A>T
18	17275577	A	T	intron_variant	MODIFIER	TRNAV-UAC	transcript	pseudogene	n.220-2523687T>A
18	17275577	A	T	intron_variant	MODIFIER	FAM185A	transcript	protein_coding	c.669+378T>A
18	17283248	G	T	upstream_gene_variant	MODIFIER	CCDC146	transcript	protein_coding	c.-22257G>T
18	17283248	G	T	intron_variant	MODIFIER	TRNAF-GAA	transcript	pseudogene	n.146+5061789G>T
18	17283248	G	T	intron_variant	MODIFIER	TRNAV-UAC	transcript	pseudogene	n.220-2531358C>A
18	17283248	G	T	intron_variant	MODIFIER	FAM185A	transcript	protein_coding	c.464-446C>A
18	17338492	C	A	upstream_gene_variant	MODIFIER	FGL2	transcript	protein_coding	c.-3102G>T
18	17338492	C	A	intron_variant	MODIFIER	TRNAF-GAA	transcript	pseudogene	n.146+5117033C>A
18	17338492	C	A	intron_variant	MODIFIER	TRNAV-UAC	transcript	pseudogene	n.220-2586602G>T
18	17338492	C	A	intron_variant	MODIFIER	CCDC146	transcript	protein_coding	c.160-29608C>A
18	17338492	C	A	intron_variant	MODIFIER	CCDC146	transcript	protein_coding	c.-67-29608C>A
18	17392834	C	T	intron_variant	MODIFIER	TRNAF-GAA	transcript	pseudogene	n.146+5171375C>T
18	17392834	C	T	intron_variant	MODIFIER	TRNAV-UAC	transcript	pseudogene	n.220-2640944G>A
18	17392834	C	T	intron_variant	MODIFIER	CCDC146	transcript	protein_coding	c.1922+99C>T

18	17392834	C	T	intron_variant	MODIFIER	CCDC146	transcript	protein_coding	c.1826+99C>T
18	17392834	C	T	intron_variant	MODIFIER	CCDC146	transcript	protein_coding	c.1691+99C>T
18	17403586	C	T	downstream_gene_variant	MODIFIER	GSAP	transcript	protein_coding	c.*3946G>A
18	17403586	C	T	downstream_gene_variant	MODIFIER	GSAP	transcript	pseudogene	n.*4457G>A
18	17403586	C	T	intron_variant	MODIFIER	TRNAF-GAA	transcript	pseudogene	n.146+5182127C>T
18	17403586	C	T	intron_variant	MODIFIER	TRNAV-UAC	transcript	pseudogene	n.220-2651696G>A
18	17403586	C	T	intron_variant	MODIFIER	CCDC146	transcript	protein_coding	c.2667+503C>T
18	17403586	C	T	intron_variant	MODIFIER	CCDC146	transcript	protein_coding	c.2571+503C>T
18	17403586	C	T	intron_variant	MODIFIER	CCDC146	transcript	protein_coding	c.2436+503C>T
18	17520342	T	TCGGGATCCCTGGGTGGCGCAGCGGTTTGGCGC	intron_variant	MODIFIER	TRNAF-GAA	transcript	pseudogene	n.146+5298883_146+5298884insCGGGATCCCTGGGTGGCGCAGCGGTTTGGCGC
18	17520342	T	TCGGGATCCCTGGGTGGCGCAGCGGTTTGGCGC	intron_variant	MODIFIER	TRNAV-UAC	transcript	pseudogene	n.220-2768453_220-2768452insGCGCCAAACCGCTGCGCCACCCAGGGATCCCG
18	17555694	A	T	intron_variant	MODIFIER	TRNAF-GAA	transcript	pseudogene	n.146+5334235A>T
18	17555694	A	T	intron_variant	MODIFIER	TRNAV-UAC	transcript	pseudogene	n.220-2803804T>A
18	17555701	G	C	intron_variant	MODIFIER	TRNAF-GAA	transcript	pseudogene	n.146+5334242G>C
18	17555701	G	C	intron_variant	MODIFIER	TRNAV-UAC	transcript	pseudogene	n.220-2803811C>G
18	17718299	AGG	A	upstream_gene_variant	MODIFIER	LOC106559955	transcript	pseudogene	n.-237_-236delCC
18	17718299	AGG	A	downstream_gene_variant	MODIFIER	LOC102153599	transcript	protein_coding	c.*5082_*5083delCC
18	17718299	AGG	A	intron_variant	MODIFIER	TRNAF-GAA	transcript	pseudogene	n.146+5496841_146+5496842delGG
18	18135529	TTATTTATTTATG	T	intron_variant	MODIFIER	TRNAF-GAA	transcript	pseudogene	n.146+5914071_146+5914082delTATTTATTTATG
18	18135529	TTATTTATTTATG	T	intron_variant	MODIFIER	TRNAV-UAC	transcript	pseudogene	n.220-3383651_220-3383640delCATAAATAAATA
18	18135529	TTATTTATTTATG	T	intron_variant	MODIFIER	MAGI2	transcript	protein_coding	c.2043+439_2043+450delCATAAATAAATA
18	18413670	GAC	G	intron_variant	MODIFIER	TRNAF-GAA	transcript	pseudogene	n.146+6192212_146+6192213delAC
18	18413670	GAC	G	intron_variant	MODIFIER	TRNAV-UAC	transcript	pseudogene	n.220-3661782_220-3661781delGT
18	18413670	GAC	G	intron_variant	MODIFIER	MAGI2	transcript	protein_coding	c.556+6318_556+6319delGT
18	18488191	AGCAG	A	intron_variant	MODIFIER	TRNAF-GAA	transcript	pseudogene	n.146+6266733_146+6266736delGCAG
18	18488191	AGCAG	A	intron_variant	MODIFIER	TRNAV-UAC	transcript	pseudogene	n.220-3736305_220-3736302delCTGC
18	18488191	AGCAG	A	intron_variant	MODIFIER	MAGI2	transcript	protein_coding	c.50-35958_50-35955delCTGC
18	18852752	G	A	intron_variant	MODIFIER	TRNAF-GAA	transcript	pseudogene	n.146+6631293G>A
18	18852752	G	A	intron_variant	MODIFIER	TRNAV-UAC	transcript	pseudogene	n.220-4100862C>T
18	18857838	G	A	intron_variant	MODIFIER	TRNAF-GAA	transcript	pseudogene	n.146+6636379G>A
18	18857838	G	A	intron_variant	MODIFIER	TRNAV-UAC	transcript	pseudogene	n.220-4105948C>T
18	18919135	T	C	intron_variant	MODIFIER	TRNAF-GAA	transcript	pseudogene	n.146+6697676T>C
18	18919135	T	C	intron_variant	MODIFIER	TRNAV-UAC	transcript	pseudogene	n.220-4167245A>G
18	18926758	C	T	intron_variant	MODIFIER	TRNAF-GAA	transcript	pseudogene	n.146+6705299C>T
18	18926758	C	T	intron_variant	MODIFIER	TRNAV-UAC	transcript	pseudogene	n.220-4174868G>A
18	18981949	G	A	intron_variant	MODIFIER	TRNAF-GAA	transcript	pseudogene	n.146+6760490G>A
18	18981949	G	A	intron_variant	MODIFIER	TRNAV-UAC	transcript	pseudogene	n.220-4230059C>T
18	19085237	A	G	intron_variant	MODIFIER	TRNAF-GAA	transcript	pseudogene	n.147-6730980A>G
18	19085237	A	G	intron_variant	MODIFIER	TRNAV-UAC	transcript	pseudogene	n.220-4333347T>C
18	19087597	A	T	intron_variant	MODIFIER	TRNAF-GAA	transcript	pseudogene	n.147-6728620A>T
18	19087597	A	T	intron_variant	MODIFIER	TRNAV-UAC	transcript	pseudogene	n.220-4335707T>A
18	19597546	T	TCGGGATCCCTGGGTGGCGCAGC	intron_variant	MODIFIER	TRNAF-GAA	transcript	pseudogene	n.147-6218671_147-6218670insCGGGATCCCTGGGTGGCGCAGC
18	19597546	T	TCGGGATCCCTGGGTGGCGCAGC	intron_variant	MODIFIER	TRNAV-UAC	transcript	pseudogene	n.220-4845657_220-4845656insGCTGCGCCACCCAGGGATCCCG
18	19773029	A	C	intron_variant	MODIFIER	TRNAF-GAA	transcript	pseudogene	n.147-6043188A>C
18	19773029	A	C	intron_variant	MODIFIER	TRNAV-UAC	transcript	pseudogene	n.219+4798110T>G

Table S4. Phenotypes and *NAPEPLD* genotypes of Rottweilers with detailed records.

Case no	LabID	Sex	Affection status (1: control; 2: case)	Age of onset of clinical signs (years)	Gait abnormalities	MRI	Necropsy	GWAS	*NAPEPLD* : CFA18:g.16987327_16987328insC	Reference
R1	A9-21140	m	2	1.75	yes	yes	(spinal cord, brainstem, cerebellum, midbrain, hippocampus, thalamus, cortex)		DD	Eagleson J.S. *et al*. (2013) *J Am Anim Hosp Assoc*. **49,** 255-261.
R2	RO002	m	2	2.3	yes	yes	(spinal cord)	X	DD	Hirschvogel K. *et al*. (2013) *BMC Vet Res*. **9,** 57.
R3	RO003	m	2	1.5	yes	no	(spinal cord)	X	DD	
R4	16255	f	2	1.5	yes	no	not available	X	DD	
	Rtw_19408	f	1					X	NN	
	Rtw_9753	m	1					X	NN	
	Rtw_17703	f	1					X	NN	
	Rtw_15735	f	1					X	NN	
	Rtw_7534	f	1					X	NN	
	Rtw_5170	m	1					X	NN	
	Rtw_GT346	f	1					X		Vaysse, A. *et al.* (2011) *PLoS Genet.* **7,** e1002316.
	Rtw_GT347	m	1					X		Vaysse, A. *et al.* (2011) *PLoS Genet.* **7,** e1002316.
	Rtw_GT348	f	1					X		Vaysse, A. *et al.* (2011) *PLoS Genet.* **7,** e1002316.
	Rtw_GT349	m	1					X		Vaysse, A. *et al.* (2011) *PLoS Genet.* **7,** e1002316.
	Rtw_GT350	f	1					X		Vaysse, A. *et al.* (2011) *PLoS Genet.* **7,** e1002316.
	Rtw_GT351	m	1					X		Vaysse, A. *et al.* (2011) *PLoS Genet.* **7,** e1002316.
	Rtw_GT352	m	1					X		Vaysse, A. *et al.* (2011) *PLoS Genet.* **7,** e1002316.
	Rtw_GT353	m	1					X		Vaysse, A. *et al.* (2011) *PLoS Genet.* **7,** e1002316.
	Rtw_GT354	m	1					X		Vaysse, A. *et al.* (2011) *PLoS Genet.* **7,** e1002316.
	Rtw_GT355	m	1					X		Vaysse, A. *et al.* (2011) *PLoS Genet.* **7,** e1002316.
	Rtw_GT356	f	1					X		Vaysse, A. *et al.* (2011) *PLoS Genet.* **7,** e1002316.
	Rtw_GT357	m	1					X		Vaysse, A. *et al.* (2011) *PLoS Genet.* **7,** e1002316.
	ROTT_BI_10510	m	1					X		Karlsson, E. *et al.* (2013) *Genome Biol.* **14,** R132.
	ROTT_BI_11267	m	1					X		Karlsson, E. *et al.* (2013) *Genome Biol.* **14,** R132.
	ROTT_BI_11268	f	1					X		Karlsson, E. *et al.* (2013) *Genome Biol.* **14,** R132.
	ROTT_BI_12433	f	1					X		Karlsson, E. *et al.* (2013) *Genome Biol.* **14,** R132.

	ROTT_BI_14555	f	1					X		Karlsson, E. *et al.* (2013) *Genome Biol.* **14,** R132.
	ROTT_BI_14563	f	1					X		Karlsson, E. *et al.* (2013) *Genome Biol.* **14,** R132.
	ROTT_BI_14565	f	1					X		Karlsson, E. *et al.* (2013) *Genome Biol.* **14,** R132.
	ROTT_BI_14571	f	1					X		Karlsson, E. *et al.* (2013) *Genome Biol.* **14,** R132.
	ROTT_BI_14572	f	1					X		Karlsson, E. *et al.* (2013) *Genome Biol.* **14,** R132.
	ROTT_BI_14574	m	1					X		Karlsson, E. *et al.* (2013) *Genome Biol.* **14,** R132.
	ROTT_BI_14575	f	1					X		Karlsson, E. *et al.* (2013) *Genome Biol.* **14,** R132.
	ROTT_BI_14581	m	1					X		Karlsson, E. *et al.* (2013) *Genome Biol.* **14,** R132.
	ROTT_BI_14585	m	1					X		Karlsson, E. *et al.* (2013) *Genome Biol.* **14,** R132.
	ROTT_BI_21187	f	1					X		Karlsson, E. *et al.* (2013) *Genome Biol.* **14,** R132.
	ROTT_BI_21845	f	1					X		Karlsson, E. *et al.* (2013) *Genome Biol.* **14,** R132.
	ROTT_BI_24125	m	1					X		Karlsson, E. *et al.* (2013) *Genome Biol.* **14,** R132.
	ROTT_BI_24126	m	1					X		Karlsson, E. *et al.* (2013) *Genome Biol.* **14,** R132.
	ROTT_BI_24713	m	1					X		Karlsson, E. *et al.* (2013) *Genome Biol.* **14,** R132.
	ROTT_BI_24845	m	1					X		Karlsson, E. *et al.* (2013) *Genome Biol.* **14,** R132.
	ROTT_BI_24846	f	1					X		Karlsson, E. *et al.* (2013) *Genome Biol.* **14,** R132.
	ROTT_BI_25314	f	1					X		Karlsson, E. *et al.* (2013) *Genome Biol.* **14,** R132.
	ROTT_BI_25858	m	1					X		Karlsson, E. *et al.* (2013) *Genome Biol.* **14,** R132.
	ROTT_BI_26164	m	1					X		Karlsson, E. *et al.* (2013) *Genome Biol.* **14,** R132.
	ROTT_BI_26274	m	1					X		Karlsson, E. *et al.* (2013) *Genome Biol.* **14,** R132.
	ROTT_BI_26275	f	1					X		Karlsson, E. *et al.* (2013) *Genome Biol.* **14,** R132.
	ROTT_BI_26278	m	1					X		Karlsson, E. *et al.* (2013) *Genome Biol.* **14,** R132.
	ROTT_BI_26279	m	1					X		Karlsson, E. *et al.* (2013) *Genome Biol.* **14,** R132.
	ROTT_BI_26280	m	1					X		Karlsson, E. *et al.* (2013) *Genome Biol.* **14,** R132.
	ROTT_BI_26282	f	1					X		Karlsson, E. *et al.* (2013) *Genome Biol.* **14,** R132.
	ROTT_BI_26284	m	1					X		Karlsson, E. *et al.* (2013) *Genome Biol.* **14,** R132.
	ROTT_BI_26285	f	1					X		Karlsson, E. *et al.* (2013) *Genome Biol.* **14,** R132.
	ROTT_BI_26286	f	1					X		Karlsson, E. *et al.* (2013) *Genome Biol.* **14,** R132.

	ROTT_BI_26288	m	1					X		Karlsson, E. *et al.* (2013) *Genome Biol.* **14,** R132.
	ROTT_BI_26293	f	1					X		Karlsson, E. *et al.* (2013) *Genome Biol.* **14,** R132.
	ROTT_BI_26294	m	1					X		Karlsson, E. *et al.* (2013) *Genome Biol.* **14,** R132.
	ROTT_BI_26295	m	1					X		Karlsson, E. *et al.* (2013) *Genome Biol.* **14,** R132.
	ROTT_BI_26296	f	1					X		Karlsson, E. *et al.* (2013) *Genome Biol.* **14,** R132.
	ROTT_BI_26297	f	1					X		Karlsson, E. *et al.* (2013) *Genome Biol.* **14,** R132.
	ROTT_BI_26298	f	1					X		Karlsson, E. *et al.* (2013) *Genome Biol.* **14,** R132.
	ROTT_BI_26299	m	1					X		Karlsson, E. *et al.* (2013) *Genome Biol.* **14,** R132.
	ROTT_BI_262	f	1					X		Karlsson, E. *et al.* (2013) *Genome Biol.* **14,** R132.
	ROTT_BI_26300	f	1					X		Karlsson, E. *et al.* (2013) *Genome Biol.* **14,** R132.
	ROTT_BI_26301	m	1					X		Karlsson, E. *et al.* (2013) *Genome Biol.* **14,** R132.
	ROTT_BI_26302	m	1					X		Karlsson, E. *et al.* (2013) *Genome Biol.* **14,** R132.
	ROTT_BI_26303	f	1					X		Karlsson, E. *et al.* (2013) *Genome Biol.* **14,** R132.
	ROTT_BI_26304	m	1					X		Karlsson, E. *et al.* (2013) *Genome Biol.* **14,** R132.
	ROTT_BI_26305	m	1					X		Karlsson, E. *et al.* (2013) *Genome Biol.* **14,** R132.
	ROTT_BI_26306	m	1					X		Karlsson, E. *et al.* (2013) *Genome Biol.* **14,** R132.
	ROTT_BI_26307	m	1					X		Karlsson, E. *et al.* (2013) *Genome Biol.* **14,** R132.
	ROTT_BI_26310	m	1					X		Karlsson, E. *et al.* (2013) *Genome Biol.* **14,** R132.
	ROTT_BI_26311	m	1					X		Karlsson, E. *et al.* (2013) *Genome Biol.* **14,** R132.
	ROTT_BI_26312	m	1					X		Karlsson, E. *et al.* (2013) *Genome Biol.* **14,** R132.
	ROTT_BI_26313	f	1					X		Karlsson, E. *et al.* (2013) *Genome Biol.* **14,** R132.
	ROTT_BI_2989	m	1					X		Karlsson, E. *et al.* (2013) *Genome Biol.* **14,** R132.
	ROTT_BI_29967	m	1					X		Karlsson, E. *et al.* (2013) *Genome Biol.* **14,** R132.
	ROTT_BI_30643	f	1					X		Karlsson, E. *et al.* (2013) *Genome Biol.* **14,** R132.
	ROTT_BI_3253	f	1					X		Karlsson, E. *et al.* (2013) *Genome Biol.* **14,** R132.
	ROTT_BI_3254	f	1					X		Karlsson, E. *et al.* (2013) *Genome Biol.* **14,** R132.
	ROTT_BI_327	m	1					X		Karlsson, E. *et al.* (2013) *Genome Biol.* **14,** R132.
	ROTT_BI_328	m	1					X		Karlsson, E. *et al.* (2013) *Genome Biol.* **14,** R132.

	ROTT_BI_330	m	1					X		Karlsson, E. *et al.* (2013) *Genome Biol.* **14,** R132.
	ROTT_BI_33189	f	1					X		Karlsson, E. *et al.* (2013) *Genome Biol.* **14,** R132.
	ROTT_BI_331	f	1					X		Karlsson, E. *et al.* (2013) *Genome Biol.* **14,** R132.
	ROTT_BI_33276	m	1					X		Karlsson, E. *et al.* (2013) *Genome Biol.* **14,** R132.
	ROTT_BI_33278	m	1					X		Karlsson, E. *et al.* (2013) *Genome Biol.* **14,** R132.
	ROTT_BI_332	m	1					X		Karlsson, E. *et al.* (2013) *Genome Biol.* **14,** R132.
	ROTT_BI_333	m	1					X		Karlsson, E. *et al.* (2013) *Genome Biol.* **14,** R132.
	ROTT_BI_3348	m	1					X		Karlsson, E. *et al.* (2013) *Genome Biol.* **14,** R132.
	ROTT_BI_334	m	1					X		Karlsson, E. *et al.* (2013) *Genome Biol.* **14,** R132.
	ROTT_BI_338	f	1					X		Karlsson, E. *et al.* (2013) *Genome Biol.* **14,** R132.
	ROTT_BI_339	m	1					X		Karlsson, E. *et al.* (2013) *Genome Biol.* **14,** R132.
	ROTT_BI_340	m	1					X		Karlsson, E. *et al.* (2013) *Genome Biol.* **14,** R132.
	ROTT_BI_342	m	1					X		Karlsson, E. *et al.* (2013) *Genome Biol.* **14,** R132.
	ROTT_BI_34727	f	1					X		Karlsson, E. *et al.* (2013) *Genome Biol.* **14,** R132.
	ROTT_BI_34730	f	1					X		Karlsson, E. *et al.* (2013) *Genome Biol.* **14,** R132.
	ROTT_BI_3477	f	1					X		Karlsson, E. *et al.* (2013) *Genome Biol.* **14,** R132.
	ROTT_BI_3478	f	1					X		Karlsson, E. *et al.* (2013) *Genome Biol.* **14,** R132.
	ROTT_BI_3676	f	1					X		Karlsson, E. *et al.* (2013) *Genome Biol.* **14,** R132.
	ROTT_BI_3826	f	1					X		Karlsson, E. *et al.* (2013) *Genome Biol.* **14,** R132.
	ROTT_BI_389	m	1					X		Karlsson, E. *et al.* (2013) *Genome Biol.* **14,** R132.
	ROTT_BI_3921	m	1					X		Karlsson, E. *et al.* (2013) *Genome Biol.* **14,** R132.
	ROTT_BI_3923	f	1					X		Karlsson, E. *et al.* (2013) *Genome Biol.* **14,** R132.
	ROTT_BI_394	m	1					X		Karlsson, E. *et al.* (2013) *Genome Biol.* **14,** R132.
	ROTT_BI_424	f	1					X		Karlsson, E. *et al.* (2013) *Genome Biol.* **14,** R132.
	ROTT_BI_433	f	1					X		Karlsson, E. *et al.* (2013) *Genome Biol.* **14,** R132.
	ROTT_BI_440	m	1					X		Karlsson, E. *et al.* (2013) *Genome Biol.* **14,** R132.
	ROTT_BI_453	m	1					X		Karlsson, E. *et al.* (2013) *Genome Biol.* **14,** R132.
	ROTT_BI_467	f	1					X		Karlsson, E. *et al.* (2013) *Genome Biol.* **14,** R132.

	ROTT_BI_46993	f	1					X		Karlsson, E. *et al.* (2013) *Genome Biol.* **14,** R132.
	ROTT_BI_469	m	1					X		Karlsson, E. *et al.* (2013) *Genome Biol.* **14,** R132.
	ROTT_BI_478	f	1					X		Karlsson, E. *et al.* (2013) *Genome Biol.* **14,** R132.
	ROTT_BI_4924	f	1					X		Karlsson, E. *et al.* (2013) *Genome Biol.* **14,** R132.
	ROTT_BI_514	f	1					X		Karlsson, E. *et al.* (2013) *Genome Biol.* **14,** R132.
	ROTT_BI_530	f	1					X		Karlsson, E. *et al.* (2013) *Genome Biol.* **14,** R132.
	ROTT_BI_538	m	1					X		Karlsson, E. *et al.* (2013) *Genome Biol.* **14,** R132.
	ROTT_BI_541	f	1					X		Karlsson, E. *et al.* (2013) *Genome Biol.* **14,** R132.
	ROTT_BI_542	f	1					X		Karlsson, E. *et al.* (2013) *Genome Biol.* **14,** R132.
	ROTT_BI_543	m	1					X		Karlsson, E. *et al.* (2013) *Genome Biol.* **14,** R132.
	ROTT_BI_557	m	1					X		Karlsson, E. *et al.* (2013) *Genome Biol.* **14,** R132.
	ROTT_BI_59353	f	1					X		Karlsson, E. *et al.* (2013) *Genome Biol.* **14,** R132.
	ROTT_BI_596	f	1					X		Karlsson, E. *et al.* (2013) *Genome Biol.* **14,** R132.
	ROTT_BI_597	f	1					X		Karlsson, E. *et al.* (2013) *Genome Biol.* **14,** R132.
	ROTT_BI_599	f	1					X		Karlsson, E. *et al.* (2013) *Genome Biol.* **14,** R132.
	ROTT_BI_604	f	1					X		Karlsson, E. *et al.* (2013) *Genome Biol.* **14,** R132.
	ROTT_BI_605	f	1					X		Karlsson, E. *et al.* (2013) *Genome Biol.* **14,** R132.
	ROTT_BI_611	f	1					X		Karlsson, E. *et al.* (2013) *Genome Biol.* **14,** R132.
	ROTT_BI_612	f	1					X		Karlsson, E. *et al.* (2013) *Genome Biol.* **14,** R132.
	ROTT_BI_613	f	1					X		Karlsson, E. *et al.* (2013) *Genome Biol.* **14,** R132.
	ROTT_BI_614	m	1					X		Karlsson, E. *et al.* (2013) *Genome Biol.* **14,** R132.
	ROTT_BI_615	f	1					X		Karlsson, E. *et al.* (2013) *Genome Biol.* **14,** R132.
	ROTT_BI_616	m	1					X		Karlsson, E. *et al.* (2013) *Genome Biol.* **14,** R132.
	ROTT_BI_633	m	1					X		Karlsson, E. *et al.* (2013) *Genome Biol.* **14,** R132.
	ROTT_BI_6634	f	1					X		Karlsson, E. *et al.* (2013) *Genome Biol.* **14,** R132.
	ROTT_BI_6654	f	1					X		Karlsson, E. *et al.* (2013) *Genome Biol.* **14,** R132.
	ROTT_BI_715	m	1					X		Karlsson, E. *et al.* (2013) *Genome Biol.* **14,** R132.
	ROTT_BI_722	m	1					X		Karlsson, E. *et al.* (2013) *Genome Biol.* **14,** R132.

	ROTT_BI_723	f	1					X		Karlsson, E. *et al.* (2013) *Genome Biol.* **14,** R132.
	ROTT_BI_724	f	1					X		Karlsson, E. *et al.* (2013) *Genome Biol.* **14,** R132.
	ROTT_BI_7263	f	1					X		Karlsson, E. *et al.* (2013) *Genome Biol.* **14,** R132.
	ROTT_BI_726	m	1					X		Karlsson, E. *et al.* (2013) *Genome Biol.* **14,** R132.
	ROTT_BI_7368	f	1					X		Karlsson, E. *et al.* (2013) *Genome Biol.* **14,** R132.
	ROTT_BI_737	m	1					X		Karlsson, E. *et al.* (2013) *Genome Biol.* **14,** R132.
	ROTT_BI_740	f	1					X		Karlsson, E. *et al.* (2013) *Genome Biol.* **14,** R132.
	ROTT_BI_751	m	1					X		Karlsson, E. *et al.* (2013) *Genome Biol.* **14,** R132.
	ROTT_BI_755	f	1					X		Karlsson, E. *et al.* (2013) *Genome Biol.* **14,** R132.
	ROTT_BI_756	m	1					X		Karlsson, E. *et al.* (2013) *Genome Biol.* **14,** R132.
	ROTT_BI_767	m	1					X		Karlsson, E. *et al.* (2013) *Genome Biol.* **14,** R132.
	ROTT_BI_770	m	1					X		Karlsson, E. *et al.* (2013) *Genome Biol.* **14,** R132.
	ROTT_BI_793	f	1					X		Karlsson, E. *et al.* (2013) *Genome Biol.* **14,** R132.
	ROTT_BI_9454	f	1					X		Karlsson, E. *et al.* (2013) *Genome Biol.* **14,** R132.
	ROTT_BI_9740	f	1					X		Karlsson, E. *et al.* (2013) *Genome Biol.* **14,** R132.
	ROTT_BI_l-457	m	1					X		Karlsson, E. *et al.* (2013) *Genome Biol.* **14,** R132.
	ROTT_BI_l1077	f	1					X		Karlsson, E. *et al.* (2013) *Genome Biol.* **14,** R132.
	ROTT_BI_l459	f	1					X		Karlsson, E. *et al.* (2013) *Genome Biol.* **14,** R132.
	ROTT_BI_ucd13	m	1					X		Karlsson, E. *et al.* (2013) *Genome Biol.* **14,** R132.

Table S5. Primers used for the characterization of the canine *NAPEPLD* gene.

Primer	Sequence	Product size	Region
NAPEPLD_F2<br>NAPEPLD_R2	CCAGGAACTCAGCCCTTCTC<br>CTGCCATCAGATCAAAGCTG	1182	exon 2
NAPEPLD_F3a<br>NAPEPLD_R3a	CTCACGTGCCAAGCAGAAC<br>TCGTAGTGGTTGTGGCTGAG	446	exon 3a
NAPEPLD_F3b<br>NAPEPLD_R3b	GGTCCTGGTGGAGATGGAC<br>CCAGCTCGCAAGTGCTTTAG	584	exon 3b
NAPEPLD_F4<br>NAPEPLD_R4	AAATGGTTTCGGTAAGTTATCAGG<br>CCTTCCTTGCCTTCTCACAG	1172	exon 4
NAPEPLD_F5<br>NAPEPLD_R5	GAGCCAGAAACCTTTCTCCAG<br>GCAGAAGTTGTTTCCCAATG	827	exon 5
NAPEPLD_I4_F1<br>NAPEPLD_I4_R1	CTGTGAGAAGGCAAGGAAGG<br>TGGAATGGATGCACATGATAC	755	intron 4
NAPEPLD_I4_F2<br>NAPEPLD_I4_R2	GTGACCTGCCCTACCATTTG<br>CATGACCATCTTCCCAATGAC	600	intron 4
NAPEPLD_I4_F3<br>NAPEPLD_I4_R3	CCTGTTCAAAGAGAAGAATCAGG<br>TGTTTCCACCAAAACCAGTG	771	intron 4
NAPEPLD_I4_F4<br>NAPEPLD_I4_R4	TTTCCTACATGATGAATTTAAGCAC<br>AAGGGAGTGACCCAGTAGGG	894	intron 4
NAPEPLD_I4_F5<br>NAPEPLD_I4_R5	TGTCTGTCCCTTTTTGGTCAC<br>AAGAGGCACAGAGAAGCAGAG	880	intron 4
NAPEPLD_I4_F6<br>NAPEPLD_I4_R6	TTTGGCTAGGTCGTGATCG<br>GGAATCGATGTGCCAAAGAC	828	intron 4
